# Enhanced Feature Selection Based on Integration Containment Neighborhoods Rough Set Approximations and Binary Honey Badger Optimization

**DOI:** 10.1155/2022/3991870

**Published:** 2022-03-10

**Authors:** Rodyna A. Hosny, Mohamed Abd Elaziz, Rehab Ali Ibrahim

**Affiliations:** ^1^Department of Mathematics, Faculty of Science, Zagazig University, Zagazig 44519, Egypt; ^2^Academy of Scientific Research and Technology (ASRT), Ajman University, Cairo, Egypt; ^3^Faculty of Computer Science & Engineering, Galala University, Suze 435611, Egypt; ^4^Artificial Intelligence Research Center (AIRC), College of Engineering and Information Technology, Ajman University, Ajman, UAE

## Abstract

This article appoints a novel model of rough set approximations (RSA), namely, rough set approximation models build on containment neighborhoods RSA (CRSA), that generalize the traditional notions of RSA and obtain valuable consequences by minifying the boundary areas. To justify this extension, it is integrated with the binary version of the honey badger optimization (HBO) algorithm as a feature selection (FS) approach. The main target of using this extension is to assess the quality of selected features. To evaluate the performance of BHBO based on CRSA, a set of ten datasets is used. In addition, the results of BHOB are compared with other well-known FS approaches. The results show the superiority of CRSA over the traditional RS approximations. In addition, they illustrate the high ability of BHBO to improve the classification accuracy overall the compared methods in terms of performance metrics.

## 1. Introduction

In recent days, the high dimensionality [1] became a big problem [2] in different fields such as human activities recognition [3], silicon-on-insulator FinFETs [4], nonlinear servo systems [5], computer vision [6], processing of IoT data [7], and feature selection. Moreover, utilizing the techniques of feature selection (FS) appeared more in many problems like the metaheuristic search techniques [8–10], for example, salp swarm algorithm (SSA) [11], grey-wolf optimization algorithm (GWO) [12], conflict monitoring optimization [13], runner-root algorithm (RRA) [14], boosting arithmetic optimization algorithm [15], electric fish-based arithmetic optimization algorithm [16], fractional calculus-based slime mould algorithm [17], and moth-flame optimization (MFO) [18]. In [19], the salp swarm algorithm was hybridized with the particle swarm optimization algorithm, where the hybridized algorithm is noted as SSAPSO, which is efficient in the procedures of exploitation and exploration. Khotimah et al. [20] hybridized the genetic algorithm (GA) with the naïve Bayes classification (NBC) to perform the exploration procedure through the classification of some incomplete data experiments. Other enhancements on other metaheuristic algorithms like the grey wolf optimization algorithm (GWO) have been performed, for example, in [21], the authors enhanced the exploration and the exploitation activity of GWO and used the new algorithm for selecting features for the galaxies images then classifying them. They utilized the opposition-based learning (OBL) and the chaotic logistic map for recommending solutions for avoiding the drawbacks of the random solutions. In addition to the above, utilizing the operators of DE with GWO as local operators improves the exploitation ability of GWO. and such hybrid solutions are updated. In addition, the disruption operator (DO) is efficient for the exploration procedure and keeps the diversity in the population of the solutions. Yet, in the data analysis problems, the uncertainty is a big problem which may cause defect in the problem solution.

Recently, the rough set theory (RS) [22, 23] has been used as an efficient tool in solving such problem. Many recent applications included utilizing RS for reducing the dimension such as feature selection [24], pattern recognition [24], and machine learning [25]. In such context, more work is in the literature review as in [26], in which the authors developed a filter feature selection method which was based on utilizing the rough set theory for identifying all class documents. A multiplication of the class by a parameter in which the documents that are proved to be existed inside is performed, and then the authors utilized the new technique for text classification. In [27], Nabwey et al. introduced using the rough set with the hypergraph for determining the relevant subset of features and utilized the proposed technique for the wart treatment prediction. In [28], Zhao et al. proposed using the rough set theory, especially the representative entropy, and the proposed method called classified nested equivalence class (CNEC) has the ability for computing the information entropy and significance for performing the feature selection. The authors tested the proposed method on the KDD Cup competition and some datasets from the UCI repository. But, the rough set theory has a drawback in dealing the feature selection that it selects only one feature in each iteration and it has a difficulty in dealing with the real-world applications which require that RS performs features discretization into many partitions. Moreover, RS is used with the metaheuristic techniques that have the ability for avoiding the RS obstacles. From the examples in the literature, in [29], the authors introduced using RS with the binary whale optimization algorithm and tested the performance on 32 datasets taken from the repository of UCI machine learning. Ibrahim et al. in [30] combined the runner-root algorithm (RRA) with RS and also with NRS and utilized the proposed technique for the galaxies images classification after selecting the relevant features. Acharjya [31] merged RS with artificial bee colony (ABC) algorithm for the application of the hepatitis disease diagnosis. Jothi [32] combined RS with the firefly-based quick reduct algorithm and used the proposed technique, RS firefly-based quick reduct (TRSFFQR), for dealing the MRI brain images. Tawhid et al. [33] combined two metaheuristic algorithms, binary particle swarm optimization and flower pollination algorithm, with RS, and then found the binary version of the combination and used it for solving some binary problems. Patra and Barman [34] proposed using RS with developing the hyperspectral band selection method for hyperspectral band selection. Patra et al. [35] presented a multiobjective FS method depending on the cultural algorithm combined with RS and compared their method with other methods. Sahlol et al. [36] proposed combining RS with the whale optimization algorithm (WOA) and used the modified algorithm for the recognition of the handwritten Arabic optical. Jothi et al. [37] combined RS with the Jaya optimization and used the modified method as feature selection method after that using it for acute lymphoblastic leukemia classification. In [38], Mafarja and Mirjalili combined the ant lion optimization (ALO) with RS for solving some classification problems. Reddy et al. [39] combined RS with the fuzzy rule for extracting the features from the dataset of the heart disease, then selecting the relevant features for performing classification. Zou et al. [40] introduced the neighborhood RS combined with the fish swarm algorithm for solving the feature selection of some datasets, where the proposed method depended also on combining the tolerance rough set (TRS) and firefly algorithm (FA). Such techniques which are used for solving feature selection problems use RS, especially elementary sets according to some classes called equivalence ones where the equivalence can be applied just for complete data, so it may be not suitable for many cases.

The issue of incomplete knowledge became a pivotal problem for several researchers, essentially in the domain of information system. There are different methods to understand indistinctness and doubtful knowledge, one of them is rough set theory (RST). The explanation of RST counts on relating one subset with two sets called upper and lower approximations that are employed to set the boundary region and accuracy degree of that subset. RST was initiated by Pawlak [41] which has been generalized by many methods as [42–54]. What interests us around those ways whose ideas that are motivated by topology, for instance, the methods of structure lower and upper approximations utilizing diverse kinds of neighborhoods, such as *z*ℛ, ℛ*z* neighborhoods [53, 55], 〈*z*〉ℛ, ℛ〈*z*〉 neighborhoods [47], and *z*ℛ*z*, ℛ〈*z*〉ℛ neighborhoods [50, 54]. Neighborhoods are significant approaches to decrease the boundary region and enhance the accuracy measure. The wish of maximizing the accuracy degree of any subset is a prime motivation factor for introducing *C*-neighborhoods system [44] that exemplifies a beneficial tool to increase the lower approximation and decrease the upper approximation compared with ℛ-neighborhoods given in [44, 46]. In addition, *C*-neighborhoods preserved most characteristics of Pawlak's approximations compared with the other systems of neighborhoods.

Furthermore, metaheuristic techniques, MHT, have more drawbacks like that more MHT depend on the evolutionary process like the evolutionary algorithms or extension for them, and also, the solutions quality is influenced by the stuck local optima. In addition, these MHT cannot solve all problems with the same efficiency according to the no free lunch theorem [56]. Therefore, this motivated us to propose an alternative FS method that depends on a recent efficient MHT called honey badger optimization (HBO) [57] algorithm that simulates the behaviour of honey badger to catch its prey. According to the mathematical model of these behaviours, HBO has been applied to solve global optimization problem and engineering problems [57]. In addition, we developed an extension of RST, named containment neighborhoods RSA (CRSA), as fitness value. According to our Knowledge, this is the first time the HBA is combined with extension rough set and used as FS method. In addition, the CRSA is a new extension of RST that is not used yet.

The HBOCRSA is used for solving the feature selection, which is initialized where the dataset is split into two parts, training and testing. After that, initialize the first set of candidate solutions by utilizing the containment neighborhoods RSA (CRSA) as a new extension of knowledge which represents a fitness function to use the training part for assessing each solution quality. Then, reaching the finest solution and using the operators of the proposed method for modernizing the current agents, as the stopping condition is met, the updating process is performed; then, obtaining the optimal solution can be used to remove the irrelevant features and evaluate the classification performance, then reducing the testing set of features.

The main contribution of this study can be summarized as follows:

1. Propose an extension of rough set approximation (RSA) named containment neighborhoods RSA (CRSA). The new extension generalizes the traditional concepts of RSA and obtains valuable consequences by minifying the boundary areas.
2. Propose a FS approach which combines the binary version of HBO (BHBO) and the new RS approximations (i.e., CRSA).
3. Assess the performance of the developed FS approach using different datasets; as well as, compare the results of well-known FS method with the results of developed method. Moreover, evaluate the performance of the competitive FS method based on CRSA with traditional RS approximation.

The rest of this paper is organized as follows: in Section 2, the basic notation about multiknowledge rough set is given.  Section 3 presents the extension of the rough set approximations based on containment neighborhoods (CRSA). In Section 4, the steps of the proposed feature selection method are introduced. Experimental results and discussion are given in Section 5. The conclusion and future work are discussed in Section 6.

## 2. Preliminaries

Let Λ be a universe (nonempty finite set), and ℛ be any relation on Λ, i.e., ℛ⊆Λ × Λ. A form (*w*, *z*) ∈ ℛ means that *w* is in relation ℛ with *z*, which is abridged as *w*ℛ*z*.

**Definition 1 .**

Let Λ be a universe. A binary relation ℛ on Λ is called [41]

1. Equivalence if it is transitive (*y*ℛ*w* whenever *y*ℛ*z* and *z*ℛ*w*), symmetric (*w*ℛ*z* if *z*ℛ*w*), and reflexive (*w*ℛ*w* for every *w* ∈ Λ)
2. Tolerance if it is both reflexive and symmetric
3. Dominance if it is both reflexive and transitive

Neighborhood systems have been adopted to characterize relationships between objects in database methods for the target of approximate retrieval.

**Definition 2 .**

Let ℛ be any binary relation on a universe Λ. Then, the ℛ-neighborhoods of *z* ∈ Λ are presented as follows:

1. Reference [55] *z*ℛ = {*y* ∈ Λ : *z*ℛ*y*}
2. Reference [55] ℛ*z* = {*y* ∈ Λ : *y*ℛ*z*}
3. Reference [54] *z*ℛ*z* = *z*ℛ∩ℛ*z*
4. Reference [47] 〈*z*〉ℛ = ∩{*y*ℛ : *z* ∈ *y*ℛ}
5. Reference [47] ℛ〈*z*〉 = ∩{ℛ*y* : *z* ∈ ℛ*y*}
6. Reference [50] ℛ〈*z*〉ℛ = 〈*z*〉ℛ∩ℛ〈*z*〉

**Proposition 1 .**

Let ℛ be any binary relation on Λ, then

1. If ℛ is a reflexive relation on Λ, then 〈*z*〉ℛ⊆*z*ℛ, ℛ〈*z*〉⊆ℛ*z* and ℛ〈*z*〉ℛ⊆*z*ℛ*z*
2. If ℛ is a symmetric relation on Λ, then *z*ℛ = ℛ*z* = *z*ℛ*z* and 〈*z*〉ℛ = ℛ〈*z*〉 = ℛ〈*z*〉ℛ

By applying *z*ℛ_*ℓ*_ neighborhoods, Abu-Donia [42] debated three categories of upper and lower approximations of any set w.r.t finite class of binary relations ℛ_*ℓ*_, *ℓ* ∈ {1,2,…, *n*}.

**Definition 3 .**

[42]. Let each ℛ_*ℓ*_, *ℓ* ∈ {1,2,…, *n*} be a binary relation on a universe Λ. If *M*⊆Λ, then the *n* lower and *n* upper approximations of *M* are given by

1. ${}_{\left[n\right]}\underline{ℛ}\left(M\right)$ = {*z* ∈ Λ : ∩_*ℓ*=1_^*n*^*z*ℛ_*ℓ*_⊆*M*}
2. ${}^{\left[n\right]}\bar{ℛ}\left(M\right)$ = {*z* ∈ Λ : (∩_*ℓ*=1_^*n*^*z*ℛ_*ℓ*_)∩*M* ≠ ∅}

Abu-Donia [43] employed the neighborhood 〈*z*〉ℛ to characterize other distinct approximations of any set w.r.t reflexive, tolerance, dominance, and equivalence relations. Furthermore, he deduced the KRA (knowledge based on the rough approximation) approach, which generalized previous RS methods

**Definition 4 .**

[43], Suppose each ℛ_*ℓ*_, *ℓ* ∈ {1,2,…, *n*} is a binary relation on a universe Λ. Then, the *n* lower and *n* upper approximations of a subset *M* of Λ are given by

1. ${}_{\left[n\right]}\underline{apr}\left(M\right)$ = {*z* ∈ Λ : ∩_*ℓ*=1_^*n*^〈*z*〉ℛ_*ℓ*_⊆*M*}
2. ${}^{\left[n\right]}\bar{apr}\left(M\right)$ = {*z* ∈ Λ : (∩_*ℓ*=1_^*n*^〈*z*〉ℛ_*ℓ*_)∩*M* ≠ ∅}

## 3. Rough Set Approximation Models Based on Containment Neighborhoods (CRSA)

**Definition 5 .**

Suppose {ℛ_*ℓ*_ : *ℓ* = 1,2,…, *n*} is a finite class of binary relations on Λ and *z* ∈ Λ. For each *ℓ*, the *C*-neighborhoods of *z* ∈ Λ are offered as follows:

1. *zC*_*ℓ*_={*y* ∈ Λ : *y*ℛ_*ℓ*_⊆*z*ℛ_*ℓ*_}
2. *C*_*ℓ*_*z*={*y* ∈ Λ : ℛ_*ℓ*_*y*⊆ℛ_*ℓ*_*z*}
3. *zC*_*ℓ*_*z* = *zC*_*ℓ*_∩*C*_*ℓ*_*z*
4. 〈*z*〉*C*_*ℓ*_ = {*y* ∈ Λ : 〈*y*〉ℛ_*ℓ*_⊆〈*z*〉ℛ_*ℓ*_}
5. *C*_*ℓ*_〈*z*〉={*y* ∈ Λ : ℛ_*ℓ*_〈*y*〉⊆ℛ_*ℓ*_〈*z*〉}
6. *C*_*ℓ*_〈*z*〉*C*_*ℓ*_ = 〈*z*〉*C*_*ℓ*_∩*C*_*ℓ*_〈*z*〉

**Proposition 2 .**

Suppose {ℛ_*ℓ*_ : *ℓ* = 1,2,…, *n*} is a finite class of binary relations on Λ. If *z* ∈ Λ, then

1. For each *ℓ*, *z* ∈ *zC*_*ℓ*_, *z* ∈ *C*_*ℓ*_*z*, and *z* ∈ *zC*_*ℓ*_*z*
2. For each *ℓ*, *z* ∈ 〈*z*〉*C*_*ℓ*_, *z* ∈ *C*_*ℓ*_〈*z*〉, and *z* ∈ *C*_*ℓ*_〈*z*〉*C*_*ℓ*_

**Proof**

Direct to prove.

**Lemma 1 .**

Suppose {ℛ_*ℓ*_ : *ℓ* = 1,2,…, *n*} is a finite class of binary relations on Λ and *w*, *z* ∈ Λ. For each *ℓ*, then the following results hold:

1. *w* ∈ *zC*_*ℓ*_ iff *wC*_*ℓ*_⊆*zC*_*ℓ*_
2. *w* ∈ *C*_*ℓ*_*z* iff *C*_*ℓ*_*w*⊆*C*_*ℓ*_*z*
3. *w* ∈ *zC*_*ℓ*_*z* iff *wC*_*ℓ*_*w*⊆*zC*_*ℓ*_*z*
4. *w* ∈ 〈*z*〉*C*_*ℓ*_ iff 〈*w*〉*C*_*ℓ*_⊆〈*z*〉*C*_*ℓ*_
5. *w* ∈ *C*_*ℓ*_〈*z*〉 iff *C*_*ℓ*_〈*w*〉⊆*C*_*ℓ*_〈*z*〉
6. *w* ∈ *C*_*ℓ*_〈*z*〉*C*_*ℓ*_ iff *C*_*ℓ*_〈*w*〉*C*_*ℓ*_⊆*C*_*ℓ*_〈*z*〉*C*_*ℓ*_

**Proof**

We shall prove (1) and the else outcomes are similar.

Let *ℓ*∈{1,2,…, *n*} and *y*∈*wC*_*ℓ*_. Then, *y*ℛ_*ℓ*_⊆*w*ℛ_*ℓ*_. Since *w*∈*zC*_*ℓ*_, then *w*ℛ_*ℓ*_⊆*z*ℛ_*ℓ*_. Hence, *y*ℛ_*ℓ*_⊆*z*ℛ_*ℓ*_ and so *y*∈*zC*_*ℓ*_, i.e., *wC*_*ℓ*_⊆*zC*_*ℓ*_, for each *ℓ*. The other side, in view of Proposition 2, *w* ∈ *wC*_*ℓ*_ for each *ℓ*. Since *wC*_*ℓ*_⊆*zC*_*ℓ*_, then *w* ∈ *zC*_*ℓ*_.

**Corollary 1 .**

1. *w* ∈ ∩_*ℓ*=1_^*n*^(*zC*_*ℓ*_) iff ∩_*ℓ*=1_^*n*^(*wC*_*ℓ*_)⊆∩_*ℓ*=1_^*n*^(*zC*_*ℓ*_)
2. *w* ∈ ∩_*ℓ*=1_^*n*^(*C*_*ℓ*_*z*) iff ∩_*ℓ*=1_^*n*^(*C*_*ℓ*_*w*)⊆∩_*ℓ*=1_^*n*^(*C*_*ℓ*_*z*)
3. *w* ∈ ∩_*ℓ*=1_^*n*^(*zC*_*ℓ*_*z*) iff ∩_*ℓ*=1_^*n*^(*wC*_*ℓ*_*w*)⊆∩_*ℓ*=1_^*n*^(*zC*_*ℓ*_*z*)
4. *w* ∈ ∩_*ℓ*=1_^*n*^(〈*z*〉*C*_*ℓ*_) iff ∩_*ℓ*=1_^*n*^(〈*w*〉*C*_*ℓ*_)⊆∩_*ℓ*=1_^*n*^(〈*z*〉*C*_*ℓ*_)
5. *w* ∈ ∩_*ℓ*=1_^*n*^(*C*_*ℓ*_〈*z*〉) iff ∩_*ℓ*=1_^*n*^(*C*_*ℓ*_〈*w*〉)⊆∩_*ℓ*=1_^*n*^(*C*_*ℓ*_〈*z*〉)
6. *w* ∈ ∩_*ℓ*=1_^*n*^(*C*_*ℓ*_〈*z*〉*C*_*ℓ*_) iff ∩_*ℓ*=1_^*n*^(*C*_*ℓ*_〈*w*〉*C*_*ℓ*_)⊆∩_*ℓ*=1_^*n*^(*C*_*ℓ*_〈*z*〉*C*_*ℓ*_)

**Proposition 3 .**

Suppose {ℛ_*ℓ*_ : *ℓ* = 1,2,…, *n*} is a finite class of reflexive relations on Λ and *z* ∈ Λ. Then, for each *ℓ*, the following results hold:

1. *zC*_*ℓ*_⊆*z*ℛ_*ℓ*_
2. *C*_*ℓ*_*z*⊆ℛ_*ℓ*_*z*
3. *zC*_*ℓ*_*z*⊆*z*ℛ_*ℓ*_*z*
4. 〈*z*〉*C*_*ℓ*_⊆〈*z*〉ℛ_*ℓ*_
5. *C*_*ℓ*_〈*z*〉⊆ℛ_*ℓ*_〈*z*〉
6. *C*_*ℓ*_〈*z*〉*C*_*ℓ*_⊆ℛ_*ℓ*_〈*z*〉ℛ_*ℓ*_

**Proof**

We prove (1). Let *w*∈*zC*_*ℓ*_, for every *ℓ*∈{1,2,…, *n*}. Hence, *w*ℛ_*ℓ*_ ⊆ *z*ℛ_*ℓ*_, for any *ℓ* ∈ {1,2,…, *n*}. Since every ℛ_*ℓ*_, *ℓ* ∈ {1,2,…, *n*} is a reflexive relation, then *w* ∈ *w*ℛ_*ℓ*_ so that *w* ∈ *z*ℛ_*ℓ*_, i.e., *zC*_*ℓ*_⊆*z*ℛ_*ℓ*_. Analogously, we can prove other consequences.

**Corollary 2 .**

Consider {ℛ_*ℓ*_ : *ℓ* = 1,2,…, *n*} is a finite collection of reflexive relations on a universe Λ and *z* ∈ Λ. Then, the following results hold:

1. ∩_*ℓ*=1_^*n*^*zC*_*ℓ*_⊆∩_*ℓ*=1_^*n*^*z*ℛ_*ℓ*_
2. ∩_*ℓ*=1_^*n*^*C*_*ℓ*_*z*⊆∩_*ℓ*=1_^*n*^ℛ_*ℓ*_*z*
3. ∩_*ℓ*=1_^*n*^*zC*_*ℓ*_*z*⊆∩_*ℓ*=1_^*n*^*z*ℛ_*ℓ*_*z*
4. ∩_*ℓ*=1_^*n*^〈*z*〉*C*_*ℓ*_⊆∩_*ℓ*=1_^*n*^〈*z*〉ℛ_*ℓ*_
5. ∩_*ℓ*=1_^*n*^*C*_*ℓ*_〈*z*〉⊆∩_*ℓ*=1_^*n*^ℛ_*ℓ*_〈*z*〉
6. ∩_*ℓ*=1_^*n*^*C*_*ℓ*_〈*z*〉*C*_*ℓ*_⊆∩_*ℓ*=1_^*n*^ℛ_*ℓ*_〈*z*〉ℛ_*ℓ*_

**Example 1 .**

If Λ = {*β*_1_, *β*_2_, *β*_3_, *β*_4_} and △ is an identity relation on Λ, suppose ℛ_1_, ℛ_2_ are two reflexive relations on Λ defined as ℛ_1_ = △∪{(*β*_1_, *β*_2_), (*β*_3_, *β*_1_), (*β*_3_, *β*_4_), (*β*_4_, *β*_3_)} and ℛ_2_ = △∪{(*β*_1_, *β*_2_), (*β*_2_, *β*_3_), (*β*_2_, *β*_4_), (*β*_3_, *β*_2_), (*β*_3_, *β*_4_), (*β*_4_, *β*_3_)}.

  Then, *β*_1_ℛ_1_ = {*β*_1_, *β*_2_}, *β*_2_ℛ_1_ = {*β*_2_}, *β*_3_ℛ_1_ = {*β*_1_, *β*_3_, *β*_4_}, *β*_4_ℛ_1_ = {*β*_3_, *β*_4_}
 
ℛ_1_*β*_1_ = {*β*_1_, *β*_3_}, ℛ_1_*β*_2_ = {*β*_1_, *β*_2_}, ℛ_1_*β*_3_ = {*β*_3_, *β*_4_}, ℛ_1_*β*_4_ = {*β*_3_, *β*_4_}
 
*β*_1_ℛ_1_*β*_1_ = {*β*_1_}, *β*_2_ℛ_1_*β*_2_ = {*β*_2_}, *β*_3_ℛ_1_*β*_3_ = {*β*_3_, *β*_4_}, *β*_4_ℛ_1_*β*_4_ = {*β*_3_, *β*_4_}
 
〈*β*_1_〉ℛ_1_ = {*β*_1_}, 〈*β*_2_〉ℛ_1_ = {*β*_2_}, 〈*β*_3_〉ℛ_1_ = {*β*_3_, *β*_4_}, 〈*β*_4_〉ℛ_1_ = {*β*_3_, *β*_4_}
 
ℛ_1_〈*β*_1_〉 = {*β*_1_}, ℛ_1_〈*β*_2_〉 = {*β*_1_, *β*_2_}, ℛ_1_〈*β*_3_〉 = {*β*_3_}, ℛ_1_〈*β*_4_〉 = {*β*_3_, *β*_4_}
 
ℛ_1_〈*β*_1_〉ℛ_1_ = {*β*_1_}, ℛ_1_〈*β*_2_〉ℛ_1_ = {*β*_2_}, ℛ_1_〈*β*_3_〉ℛ_1_= {*β*_3_}, ℛ_1_〈*β*_4_〉ℛ_1_ = {*β*_3_, *β*_4_}
 
*β*_1_*C*_1_ = {*β*_1_, *β*_2_}, *β*_2_*C*_1_ = {*β*_2_}, *β*_3_*C*_1_ = {*β*_3_, *β*_4_}, *β*_4_*C*_1_ = {*β*_4_}
 
*C*_1_*β*_1_ = {*β*_1_}, *C*_1_*β*_2_ = {*β*_2_}, *C*_1_*β*_3_ = {*β*_3_, *β*_4_}, *C*_1_*β*_4_ = {*β*_3_, *β*_4_}
 
*β*_1_*C*_1_*β*_1_ = {*β*_1_}, *β*_2_*C*_1_*β*_2_ = {*β*_2_}, *β*_3_*C*_1_*β*_3_ = {*β*_3_, *β*_4_}, *β*_4_*C*_1_*β*_4_ = {*β*_4_}
 
〈*β*_1_〉*C*_1_ = {*β*_1_}, 〈*β*_2_〉*C*_1_ = {*β*_2_}, 〈*β*_3_〉*C*_1_ = {*β*_3_, *β*_4_}, 〈*β*_4_〉*C*_1_ = {*β*_3_, *β*_4_}
 
*C*_1_〈*β*_1_〉 = {*β*_1_}, *C*_1_〈*β*_2_〉 = {*β*_1_, *β*_2_}, *C*_1_〈*β*_3_〉 = {*β*_3_}, *C*_1_〈*β*_4_〉 = {*β*_3_, *β*_4_}
 
*C*_1_〈*β*_1_〉*C*_1_ = {*β*_1_}, *C*_1_〈*β*_2_〉*C*_1_ = {*β*_2_}, *C*_1_〈*β*_3_〉*C*_1_ = {*β*_3_}, *C*_1_〈*β*_4_〉*C*_1_ = {*β*_3_, *β*_4_}
 
*β*_1_ℛ_2_ = {*β*_1_, *β*_2_}, *β*_2_ℛ_2_ = {*β*_2_, *β*_3_, *β*_4_}, *β*_3_ℛ_2_ = {*β*_2_, *β*_3_, *β*_4_}, *β*_4_ℛ_2_ = {*β*_3_, *β*_4_}
 
ℛ_2_*β*_1_ = {*β*_1_}, ℛ_2_*β*_2_ = {*β*_1_, *β*_2_, *β*_3_}, ℛ_2_*β*_3_ = {*β*_2_, *β*_3_, *β*_4_}, ℛ_2_*β*_4_ = {*β*_2_, *β*_3_, *β*_4_}
 
*β*_1_ℛ_2_*β*_1_ = {*β*_1_}, *β*_2_ℛ_2_*β*_2_ = {*β*_2_, *β*_3_}, *β*_3_ℛ_2_*β*_3_ = {*β*_2_, *β*_3_, *β*_4_}, *β*_4_ℛ_2_*β*_4_ = {*β*_3_, *β*_4_}
 
〈*β*_1_〉ℛ_2_ = {*β*_1_, *β*_2_}, 〈*β*_2_〉ℛ_2_ = {*β*_2_}, 〈*β*_3_〉ℛ_2_ = {*β*_3_, *β*_4_}, 〈*β*_4_〉ℛ_2_ = {*β*_3_, *β*_4_}
 
ℛ_2_〈*β*_1_〉 = {*β*_1_}, ℛ_2_〈*β*_2_〉 = {*β*_2_, *β*_3_}, ℛ_2_〈*β*_3_〉 = {*β*_2_, *β*_3_}, ℛ_2_〈*β*_4_〉 = {*β*_2_, *β*_3_, *β*_4_}
 
ℛ_2_〈*β*_1_〉ℛ_2_ = {*β*_1_}, ℛ_2_〈*β*_2_〉ℛ_2_ = {*β*_2_}, ℛ_2_〈*β*_3_〉ℛ_2_ = {*β*_3_}, ℛ_2_〈*β*_4_〉ℛ_2_ = {*β*_3_, *β*_4_}
 
*β*_1_*C*_2_ = {*β*_1_}, *β*_2_*C*_2_ = {*β*_2_, *β*_3_, *β*_4_}, *β*_3_*C*_2_ = {*β*_2_, *β*_3_, *β*_4_}, *β*_4_*C*_2_ = {*β*_4_}
 
*C*_2_*β*_1_ = {*β*_1_}, *C*_2_*β*_2_ = {*β*_1_, *β*_2_}, *C*_2_*β*_3_ = {*β*_3_, *β*_4_}, *C*_2_*β*_4_ = {*β*_3_, *β*_4_}
 
*β*_1_*C*_2_*β*_1_ = {*β*_1_}, *β*_2_*C*_2_*β*_2_ = {*β*_2_}, *β*_3_*C*_2_*β*_3_ = {*β*_3_, *β*_4_}, *β*_4_*C*_2_*β*_4_ = {*β*_4_}
 
〈*β*_1_〉*C*_2_ = {*β*_1_, *β*_2_}, 〈*β*_2_〉*C*_2_ = {*β*_2_}, 〈*β*_3_〉*C*_2_ = {*β*_3_, *β*_4_}, 〈*β*_4_〉*C*_2_ = {*β*_3_, *β*_4_}
 
*C*_2_〈*β*_1_〉 = {*β*_1_}, *C*_2_〈*β*_2_〉 = {*β*_2_, *β*_3_}, *C*_2_〈*β*_3_〉 = {*β*_2_, *β*_3_}, *C*_2_〈*β*_4_〉 = {*β*_2_, *β*_3_, *β*_4_}
 
*C*_2_〈*β*_1_〉*C*_2_ = {*β*_1_}, *C*_2_〈*β*_2_〉*C*_2_ = {*β*_2_}, *C*_2_〈*β*_3_〉*C*_2_ = {*β*_3_}, *C*_2_〈*β*_4_〉*C*_2_ = {*β*_3_, *β*_4_}

**Remark 1 .**

With reflexive relations ℛ_1_, ℛ_2_ on Λ, we get

1. Generally, the opposite of Proposition 3 may not be valid as we consider in example 1.
2. Generally, the opposite of Corollary 2 may not be valid as we consider in example 1, where *β*_4_ℛ_1_ = {*β*_3_, *β*_4_}, *β*_4_ℛ_2_ = {*β*_3_, *β*_4_}, *β*_4_*C*_1_ = {*β*_4_}, and *β*_4_*C*_2_ = {*β*_4_}, ∩_*ℓ*=1_^2^*β*_4_ℛ_*ℓ*_⊈∩_*ℓ*=1_^2^*β*_4_*C*_*ℓ*_.
3. If *ℓ*∈{1,2}, then the concepts of *zC*_*ℓ*_, 〈*z*〉*C*_*ℓ*_ (resp. *C*_*ℓ*_*z*, *C*_*ℓ*_〈*z*〉 and *zC*_*ℓ*_*z*, *C*_*ℓ*_〈*z*〉*C*_*ℓ*_) are incomparable. see example 1, although 〈*z*〉ℛ_*ℓ*_⊆*z*ℛ_*ℓ*_, ℛ_*ℓ*_〈*z*〉⊆ℛ_*ℓ*_*z*, and ℛ_*ℓ*_〈*z*〉ℛ_*ℓ*_⊆*z*ℛ_*ℓ*_*z*, as it is shown in Proposition 1.

**Proposition 4 .**

Assume that {ℛ_*ℓ*_ : *ℓ* = 1,2,…, *n*} is a finite collection of transitive relations on a universe Λ and *z*  ∈  Λ. Then, for each *ℓ*, the following results hold:

1. *z*ℛ_*ℓ*_⊆*zC*_*ℓ*_
2. ℛ_*ℓ*_*z*⊆*C*_*ℓ*_*z*
3. *z*ℛ_*ℓ*_*z*⊆*zC*_*ℓ*_*z*
4. 〈*z*〉ℛ_*ℓ*_⊆〈*z*〉*C*_*ℓ*_
5. ℛ_*ℓ*_〈*z*〉⊆*C*_*ℓ*_〈*z*〉
6. ℛ_*ℓ*_〈*z*〉ℛ_*ℓ*_⊆*C*_*ℓ*_〈*z*〉*C*_*ℓ*_

**Proof**

We prove (1) and the rest of the proof is similar. Let *ℓ* ∈ {1,2,…, *n*}. Suppose *w*∈*z*ℛ_*ℓ*_, then *z*ℛ_*ℓ*_*w*. We shall prove *w* ∈ *zC*_*ℓ*_, i.e., *w*ℛ_*ℓ*_⊆*z*ℛ_*ℓ*_. Let *y* ∈ *w*ℛ_*ℓ*_, then *w*ℛ_*ℓ*_*y*. Since each ℛ_*ℓ*_ is a transitive relation on Λ, then *y*∈*z*ℛ_*ℓ*_ and so *w*ℛ_*ℓ*_⊆*z*ℛ_*ℓ*_, i.e., *w*  ∈  *zC*_*ℓ*_. Consequently, *z*ℛ_*ℓ*_⊆*zC*_*ℓ*_.

**Corollary 3 .**

Assume that {ℛ_*ℓ*_ : *ℓ* = 1,2,…, *n*} is a finite collection of transitive relations on a universe Λ and *z* ∈ Λ. Then, the following results hold:

1. ∩_*ℓ*=1_^*n*^*z*ℛ_*ℓ*_⊆∩_*ℓ*=1_^*n*^*zC*_*ℓ*_
2. ∩_*ℓ*=1_^*n*^ℛ_*ℓ*_*z*⊆∩_*ℓ*=1_^*n*^*C*_*ℓ*_*z*
3. ∩_*ℓ*=1_^*n*^*z*ℛ_*ℓ*_*z*⊆∩_*ℓ*=1_^*n*^*zC*_*ℓ*_*z*
4. ∩_*ℓ*=1_^*n*^〈*z*〉ℛ_*ℓ*_⊆∩_*ℓ*=1_^*n*^〈*z*〉*C*_*ℓ*_
5. ∩_*ℓ*=1_^*n*^ℛ_*ℓ*_〈*z*〉⊆∩_*ℓ*=1_^*n*^*C*_*ℓ*_〈*z*〉
6. ∩_*ℓ*=1_^*n*^ℛ_*ℓ*_〈*z*〉ℛ_*ℓ*_⊆∩_*ℓ*=1_^*n*^*C*_*ℓ*_〈*z*〉*C*_*ℓ*_

**Example 2 .**

Let Λ = {*β*_1_, *β*_2_, *β*_3_, *β*_4_}. Suppose ℛ_1_, ℛ_2_ are two transitive relations on Λ defined as ℛ_1_ = {(*β*_1_, *β*_2_), (*β*_1_, *β*_3_), (*β*_1_, *β*_4_), (*β*_3_, *β*_2_), (*β*_3_, *β*_4_), (*β*_4_, *β*_2_)} and ℛ_2_ = {(*β*_3_, *β*_1_), (*β*_4_, *β*_1_), (*β*_4_, *β*_3_)}. Hence,

 
*β*_1_ℛ_1_ = {*β*_2_, *β*_3_, *β*_4_}, *β*_2_ℛ_1_ = ∅, *β*_3_ℛ_1_ = {*β*_2_, *β*_4_}, *β*_4_ℛ_1_ = {*β*_2_}
 
ℛ_1_*β*_1_ = ∅, ℛ_1_*β*_2_ = {*β*_1_, *β*_3_, *β*_4_}, ℛ_1_*β*_3_ = {*β*_1_}, ℛ_1_*β*_4_ = {*β*_1_, *β*_3_}
 
*β*_1_ℛ_1_*β*_1_ = ∅, *β*_2_ℛ_1_*β*_2_ = ∅, *β*_3_ℛ_1_*β*_3_ = ∅, *β*_4_ℛ_1_*β*_4_ = ∅
 
〈*β*_1_〉ℛ_1_ = ∅, 〈*β*_2_〉ℛ_1_ = {*β*_2_}, 〈*β*_3_〉ℛ_1_ = {*β*_2_, *β*_3_, *β*_4_}, 〈*β*_4_〉ℛ_1_ = {*β*_2_, *β*_4_}
 
ℛ_1_〈*β*_1_〉 = {*β*_1_}, ℛ_1_〈*β*_2_〉 = ∅, ℛ_1_〈*β*_3_〉 = {*β*_1_, *β*_3_}, ℛ_1_〈*β*_4_〉 = {*β*_1_, *β*_3_, *β*_4_}
 
ℛ_1_〈*β*_1_〉ℛ_1_ = ∅, ℛ_1_〈*β*_2_〉ℛ_1_ = ∅, ℛ_1_〈*β*_3_〉ℛ_1_ = {*β*_3_}, ℛ_1_〈*β*_4_〉ℛ_1_ = {*β*_4_}
 
*β*_1_*C*_1_ = Λ, *β*_2_*C*_1_ = {*β*_2_}, *β*_3_*C*_1_ = {*β*_2_, *β*_3_, *β*_4_}, *β*_4_*C*_1_ = {*β*_2_, *β*_4_}
 
*C*_1_*β*_1_ = {*β*_1_}, *C*_1_*β*_2_ = Λ, *C*_1_*β*_3_ = {*β*_1_, *β*_3_}, *C*_1_*β*_4_ = {*β*_1_, *β*_3_, *β*_4_}
 
*β*_1_*C*_1_*β*_1_ = {*β*_1_}, *β*_2_*C*_1_*β*_2_ = {*β*_2_}, *β*_3_*C*_1_*β*_3_ = {*β*_3_}, *β*_4_*C*_1_*β*_4_ = {*β*_4_}
 
〈*β*_1_〉*C*_1_ = {*β*_1_}, 〈*β*_2_〉*C*_1_ = {*β*_1_, *β*_2_}, 〈*β*_3_〉*C*_1_ = Λ, 〈*β*_4_〉*C*_1_ = {*β*_1_, *β*_2_, *β*_4_}
 
*C*_1_〈*β*_1_〉 = {*β*_1_, *β*_2_}, *C*_1_〈*β*_2_〉 = {*β*_2_}, *C*_1_〈*β*_3_〉 = {*β*_1_, *β*_2_, *β*_3_}, *C*_1_〈*β*_4_〉 = Λ
 
*C*_1_〈*β*_1_〉*C*_1_ = {*β*_1_}, *C*_1_〈*β*_2_〉*C*_1_ = {*β*_2_}, *C*_1_〈*β*_3_〉*C*_1_ = {*β*_1_, *β*_2_, *β*_3_}, *C*_1_〈*β*_4_〉*C*_1_ = {*β*_1_, *β*_2_, *β*_4_}
 
*β*_1_ℛ_2_ = ∅, *β*_2_ℛ_2_ = ∅, *β*_3_ℛ_2_ = {*β*_1_}, *β*_4_ℛ_2_ = {*β*_1_, *β*_3_}
 
ℛ_2_*β*_1_ = {*β*_3_, *β*_4_}, ℛ_2_*β*_2_ = ∅, ℛ_2_*β*_3_ = {*β*_4_}, ℛ_2_*β*_4_ = ∅
 
*β*_1_ℛ_2_*β*_1_ = ∅, *β*_2_ℛ_2_*β*_2_ = ∅, *β*_3_ℛ_2_*β*_3_ = ∅, *β*_4_ℛ_2_*β*_4_ = ∅
 
〈*β*_1_〉ℛ_2_ = {*β*_1_}, 〈*β*_2_〉ℛ_2_ = ∅, 〈*β*_3_〉ℛ_2_ = {*β*_1_, *β*_3_}, 〈*β*_4_〉ℛ_2_ = ∅
 
ℛ_2_〈*β*_1_〉 = ∅, ℛ_2_〈*β*_2_〉 = ∅, ℛ_2_〈*β*_3_〉 = {*β*_3_, *β*_4_}, ℛ_2_〈*β*_4_〉 = {*β*_4_}
 
ℛ_2_〈*β*_1_〉ℛ_2_ = ∅, ℛ_2_〈*β*_2_〉ℛ_2_ = ∅, ℛ_2_〈*β*_3_〉ℛ_2_ = {*β*_3_}, ℛ_2_〈*β*_4_〉ℛ_2_ = ∅
 
*β*_1_*C*_2_ = {*β*_1_, *β*_2_}, *β*_2_*C*_2_ = {*β*_1_, *β*_2_}, *β*_3_*C*_2_ = {*β*_1_, *β*_2_, *β*_3_}, *β*_4_*C*_2_ = Λ
 
*C*_2_*β*_1_ = Λ, *C*_2_*β*_2_ = {*β*_2_, *β*_4_}, *C*_2_*β*_3_ = {*β*_2_, *β*_3_, *β*_4_}, *C*_2_*β*_4_ = {*β*_2_, *β*_4_}
 
*β*_1_*C*_2_*β*_1_ = {*β*_1_, *β*_2_}, *β*_2_*C*_2_*β*_2_ = {*β*_2_}, *β*_3_*C*_2_*β*_3_ = {*β*_2_, *β*_3_}, *β*_4_*C*_2_*β*_4_ = {*β*_2_, *β*_4_}
 
〈*β*_1_〉*C*_2_ = {*β*_1_, *β*_2_, *β*_4_}, 〈*β*_2_〉*C*_2_ = {*β*_2_, *β*_4_}, 〈*β*_3_〉*C*_2_ = Λ, 〈*β*_4_〉*C*_2_ = {*β*_2_, *β*_4_}
 
*C*_2_〈*β*_1_〉 = {*β*_1_, *β*_2_}, *C*_2_〈*β*_2_〉 = {*β*_1_, *β*_2_}, *C*_2_〈*β*_3_〉 = Λ, *C*_2_〈*β*_4_〉 = {*β*_1_, *β*_2_, *β*_4_}
 
*C*_2_〈*β*_1_〉*C*_2_ = {*β*_1_, *β*_2_}, *C*_2_〈*β*_2_〉*C*_2_ = {*β*_2_}, *C*_2_〈*β*_3_〉*C*_2_ = Λ, *C*_2_〈*β*_4_〉*C*_2_ = {*β*_2_, *β*_4_}

**Remark 2 .**

With transitive relations ℛ_1_, ℛ_2_ on Λ (see example 2), we have

1. If *ℓ* ∈ {1,2}, then the concepts of *z*ℛ_*ℓ*_, 〈*z*〉ℛ_*ℓ*_ (resp. ℛ_*ℓ*_*z*, ℛ_*ℓ*_〈*z*〉 and *z*ℛ_*ℓ*_*z*, ℛ_*ℓ*_〈*z*〉ℛ_*ℓ*_) are incomparable
2. If *ℓ* ∈ {1,2}, then the concepts of *zC*_*ℓ*_, 〈*z*〉*C*_*ℓ*_ (resp. *C*_*ℓ*_*z*, *C*_*ℓ*_〈*z*〉 and *zC*_*ℓ*_*z*, *C*_*ℓ*_〈*z*〉*C*_*ℓ*_) are incomparable
3. Mainly, the converse of Proposition 4 and Corollary 3 cannot be valid as we see in example 2

In view of Propositions 3 and 4, we have

**Proposition 5 .**

suppose that {ℛ_*ℓ*_ : *ℓ* = 1,2,…, *n*} is a finite class of dominance relations on Λ and *z* ∈ Λ. Then, for all *ℓ*, the following results hold:

1. *z*ℛ_*ℓ*_ = *zC*_*ℓ*_
2. ℛ_*ℓ*_*z* = *C*_*ℓ*_*z*
3. *z*ℛ_*ℓ*_*z* = *zC*_*ℓ*_*z*
4. 〈*z*〉ℛ_*ℓ*_ = 〈*z*〉*C*_*ℓ*_
5. ℛ_*ℓ*_〈*z*〉 = *C*_*ℓ*_〈*z*〉
6. ℛ_*ℓ*_〈*z*〉ℛ_*ℓ*_ = *C*_*ℓ*_〈*z*〉*C*_*ℓ*_

**Corollary 4 .**

Assume that {ℛ_*ℓ*_ : *ℓ* = 1,2,…, *n*} is a finite class of dominance relations on Λ and *z* ∈ Λ. Then,

1. ∩_*ℓ*=1_^*n*^*z*ℛ_*ℓ*_ = ∩_*ℓ*=1_^*n*^*zC*_*ℓ*_
2. ∩_*ℓ*=1_^*n*^ℛ_*ℓ*_*z* = ∩_*ℓ*=1_^*n*^*C*_*ℓ*_*z*
3. ∩_*ℓ*=1_^*n*^*z*ℛ_*ℓ*_*z* = ∩_*ℓ*=1_^*n*^*zC*_*ℓ*_*z*
4. ∩_*ℓ*=1_^*n*^〈*z*〉ℛ_*ℓ*_ = ∩_*ℓ*=1_^*n*^〈*z*〉*C*_*ℓ*_
5. ∩_*ℓ*=1_^*n*^ℛ_*ℓ*_〈*z*〉 = ∩_*ℓ*=1_^*n*^*C*_*ℓ*_〈*z*〉
6. ∩_*ℓ*=1_^*n*^ℛ_*ℓ*_〈*z*〉ℛ_*ℓ*_ = ∩_*ℓ*=1_^*n*^*C*_*ℓ*_〈*z*〉*C*_*ℓ*_

**Proposition 6 .**

Suppose that {ℛ_*ℓ*_ : *ℓ* = 1,2,…, *n*} is a finite collection of dominance relations on Λ and *x*, *y* ∈ Λ. Then, for each *ℓ*, *y* ∈ *x*ℛ_*ℓ*_*x* iff *y*ℛ_*ℓ*_*y* = *x*ℛ_*ℓ*_*x*.

**Proof**

(⟹) Let *y*∈*x*ℛ_*ℓ*_*x* and *z*∈*y*ℛ_*ℓ*_*y*, then *y*∈*x*ℛ_*ℓ*_, *y*∈ℛ_*ℓ*_*x* and *z*∈*y*ℛ_*ℓ*_, *z* ∈ ℛ_*ℓ*_*y*, i.e., *x*ℛ_*ℓ*_*y*, *y*ℛ_*ℓ*_*x*, *y*ℛ_*ℓ*_*z*, and *z*ℛ_*ℓ*_*y*. Since each relation is transitive, then *x*ℛ_*ℓ*_*z* and *z*ℛ_*ℓ*_*x*, i.e., *z* ∈ *x*ℛ_*ℓ*_*x*. Hence, *y*ℛ_*ℓ*_*y*⊆*x*ℛ_*ℓ*_*x*. Also, if *y*∈*x*ℛ_*ℓ*_*x* and *z*∈*x*ℛ_*ℓ*_*x*, then *y*∈*x*ℛ_*ℓ*_, *y*∈ℛ_*ℓ*_*x* and *z*∈*x*ℛ_*ℓ*_, *z*∈ℛ_*ℓ*_*x*. Since each relation is transitive, then *z* ∈ *y*ℛ_*ℓ*_*y* and so *x*ℛ_*ℓ*_*x*⊆*y*ℛ_*ℓ*_*y*. Consequently, *y*ℛ_*ℓ*_*y* = *x*ℛ_*ℓ*_*x* for every *y* ∈ *x*ℛ_*ℓ*_*x*.

(⟸) Suppose *y*ℛ_*ℓ*_*y* = *x*ℛ_*ℓ*_*x*. Since each relation is reflexive, then *y* ∈ *x*ℛ_*ℓ*_*x*.

In view of (3) of Proposition 5, the following corollary holds.

**Corollary 5 .**

If {ℛ_*ℓ*_ : *ℓ* = 1,2,…, *n*} is a finite class of dominance relations on a universe Λ and *x*, *y* ∈ Λ, then for all *ℓ*, *y* ∈ *xC*_*ℓ*_*x* iff *yC*_*ℓ*_*y* = *xC*_*ℓ*_*x*.

**Remark 3 .**

Suppose Λ is a universe. If {ℛ_*ℓ*_ : *ℓ*  =  1,2,…, *n*} represents class of reflexive (resp. transitive) relations, then Proposition 5 will not be correct, as shown in Examples 1 and 2.

**Proposition 7 .**

If {ℛ_*ℓ*_ : *ℓ* = 1,2,…, *n*} is a finite collection of symmetric relations on Λ and *z* ∈ Λ, then *zC*_*ℓ*_ =  *C*_*ℓ*_*z* =  *zC*_*ℓ*_*z* and 〈*z*〉*C*_*ℓ*_ =  *C*_*ℓ*_〈*z*〉 =  *C*_*ℓ*_〈*z*〉*C*_*ℓ*_, for all *ℓ*.

**Proof**

Straightforward.

Depending on containment neighborhoods generated from the collection of finite binary relations, we propose in the next part some new sorts of lower and upper approximations.

**Definition 6 .**

Suppose that every ℛ_*ℓ*_, *ℓ* ∈ {1,2,…, *n*} is a relation on Λ and *z* ∈ Λ. Based on containment neighborhoods *zC*_*ℓ*_*z* = *zC*_*ℓ*_∩*C*_*ℓ*_*z*, *ℓ* ∈ {1,2,…, *n*}, the pair (_(*n*)_*E*_⊗_*L*(*M*),^(*n*)^*E*_⊗_*U*(*M*)) stands for lower and upper approximations of a set *M*, respectively, defined as the following:

(i) The *n*-*E*_⊗_ lower approx. operator of *M* is
  (1) $$\begin{array}{c} {}_{\left(n\right)}E_{⊗}L\left(M\right)=\left\{z\in \Lambda :\cap_{\ell =1}^{n}zC_{\ell }z\subseteq M\right\}. \end{array}$$
(ii) The *n*-*E*_⊗_ upper approx. operator of *M* is
  (2) $$\begin{array}{c} {}^{\left(n\right)}E_{⊗}U\left(M\right)=\left\{z\in \Lambda :\cap_{\ell =1}^{n}\left(zC_{\ell }z\right)\cap M\neq \emptyset \right\}. \end{array}$$
(iii) The *n*-*E*_⊗_ boundary of *M* is
  (3) $$\begin{array}{c} nE_{⊗}B\left(M\right){=}^{\left(n\right)}E_{⊗}U\left(M\right){-}_{\left(n\right)}E_{⊗}L\left(M\right). \end{array}$$
(iv) The *n*-*E*_⊗_ accuracy measure of any set *M* is
  (4) $$\begin{array}{c} {}_{\left(n\right)}\alpha^{E_{⊗}}\left(M\right)=\frac{\left|{}_{\left(n\right)}E_{⊗}L\left(M\right)\right|}{\left|{}^{\left(n\right)}E_{⊗}U\left(M\right)\right|}, \end{array}$$

where the cardinality of any set is denoted by the symbol |.|.

A set *M* is called definable if _(*n*)_*E*_⊗_*L*(*M*) = ^(*n*)^*E*_⊗_*U*(*M*), and it is rough set otherwise.

Our model acquires whole essential properties of the original rough set model, thus various main characteristics of the *n*-*E*_⊗_ lower and *n*-*E*_⊗_ upper operators in the next proposition are inserted.

**Proposition 8 .**

Suppose that every ℛ_*ℓ*_ is a binary relation on a universe Λ, *ℓ* ∈ {1,2,…, *n*}. If $M,\overset{´}{M}\subseteq \Lambda$, so the next conditions hold:

1. _(*n*)_*E*_⊗_*L*(*M*) = (^(*n*)^*E*_⊗_*U*(*M*^*c*^))^*c*^
2. _(*n*)_*E*_⊗_*L*(Λ) = Λ
3. ${}_{\left(n\right)}E_{⊗}L\left(M\cap \overset{´}{M}\right)$ = ${}_{\left(n\right)}E_{⊗}L\left(M\right)\cap_{\left(n\right)}E_{⊗}L\left(\overset{´}{M}\right)$
4. ${}_{\left(n\right)}E_{⊗}L\left(M\cup \overset{´}{M}\right){⊇}_{\left(n\right)}E_{⊗}L\left(M\right)\cup_{\left(n\right)}E_{⊗}L\left(\overset{´}{M}\right)$
5. $M\subseteq \overset{´}{M}\Rightarrow_{\left(n\right)}E_{⊗}L\left(M\right)\subseteq_{\left(n\right)}E_{⊗}L\left(\overset{´}{M}\right)$
6. _(*n*)_*E*_⊗_*L*(∅) = ∅
7. _(*n*)_*E*_⊗_*L*(*M*)⊆*M*
8. _(*n*)_*E*_⊗_*L*(*M*) = _(*n*)_*E*_⊗_*L*(_(*n*)_*E*_⊗_*L*(*M*))
9. ^(*n*)^*E*_⊗_*U*(*M*) = (_(*n*)_*E*_⊗_*L*(*M*^*c*^))^*c*^
10. ^(*n*)^*E*_⊗_*U*(∅) = ∅
11. ${}^{\left(n\right)}E_{⊗}U\left(M\cup \overset{´}{M}\right)$ = ${}^{\left(n\right)}E_{⊗}U\left(M\right)\cup^{\left(n\right)}E_{⊗}U\left(\overset{´}{M}\right)$
12. ${}^{\left(n\right)}E_{⊗}U\left(M\cap \overset{´}{M}\right)\subseteq^{\left(n\right)}E_{⊗}U\left(M\right)\cap^{\left(n\right)}E_{⊗}U\left(\overset{´}{M}\right)$
13. $M\subseteq \overset{´}{M}\Rightarrow^{\left(n\right)}E_{⊗}U\left(\left(M\right)\right)\subseteq^{\left(n\right)}E_{⊗}U\left(\overset{´}{M}\right)$
14. ^(*n*)^*E*_⊗_*U*(Λ) = Λ
15. *M*⊆^(*n*)^*E*_⊗_*U*(*M*)
16. ^(*n*)^*E*_⊗_*U*(*M*) = ^(*n*)^*E*_⊗_*U*(^(*n*)^*E*_⊗_*U*(*M*))

**Proof**

The characteristics of _(*n*)_*E*_⊗_*L*(.) and ^(*n*)^*E*_⊗_*U*(.) operators are dual, then we will examine one of them.

Statements 2, 4, and 6 are obvious.

1. (^(*n*)^*E*_⊗_*U*(*M*^*c*^))^*c*^ =  [{*z* ∈ Λ : (∩_*ℓ*=1_^*n*^(*zC*_*ℓ*_*z*))∩*M*^*c*^ ≠ ∅}]^*c*^ = {*z* ∈ Λ : ∩_*ℓ*=1_^*n*^(*zC*_*ℓ*_*z*)⊆*M*} = _(*n*)_*E*_⊗_*L*(*M*).
2. ${}_{\left(n\right)}E_{⊗}L\left(M\cap \overset{´}{M}\right)$ = $\left\{z\in \Lambda :\cap_{\ell =1}^{n}\left(zC_{\ell }z\right)\subseteq \left(M\cap \overset{´}{M}\right)\right\}$  = $\left\{z\in \Lambda :\cap_{\ell =1}^{n}\left(zC_{\ell }z\right)\subseteq M\right\}\cap \left\{z\in \Lambda :\cap_{\ell =1}^{n}\left(zC_{\ell }z\right)\subseteq \overset{´}{M}\right\}$  = ${}_{\left(n\right)}E_{⊗}L\left(M\right)\cap_{\left(n\right)}E_{⊗}L\left(\overset{´}{M}\right)$.
3. Suppose *z*∈_(*n*)_*E*_⊗_*L*(*M*), then ∩_*ℓ*=1_^*n*^(*zC*_*ℓ*_*z*)⊆*M*. Since $M\subseteq \overset{´}{M}$, then $\cap_{\ell =1}^{n}\left(zC_{\ell }z\right)\subseteq \overset{´}{M}$ and so $z\in_{\left(n\right)}E_{⊗}L\left(\overset{´}{M}\right)$. Hence, ${}_{\left(n\right)}E_{⊗}L\left(M\right)\subseteq_{\left(n\right)}E_{⊗}L\left(\overset{´}{M}\right)$.
4. Let *z*∈_(*n*)_*E*_⊗_*L*(*M*), then ∩_*ℓ*=1_^*n*^(*zC*_*ℓ*_*z*)⊆*M*. In view of (1) of Proposition 2, then *z* ∈ ∩_*ℓ*=1_^*n*^(*zC*_*ℓ*_*z*). Hence, *z* ∈ *M*. So, _(*n*)_*E*_⊗_*L*(*M*)⊆*M*.
5. According to properties 5 and 7, we get _(*n*)_*E*_⊗_*L*(_(*n*)_*E*_⊗_*L*(*M*))⊆_(*n*)_*E*_⊗_*L*(*M*). The other side, let *z*∈_(*n*)_*E*_⊗_*L*(*M*), then ∩_*ℓ*=1_^*n*^(*zC*_*ℓ*_*z*)⊆*M*. Let *y*∈∩_*ℓ*=1_^*n*^(*zC*_*ℓ*_*z*). Hence, in view of Corollary 1, ∩_*ℓ*=1_^*n*^(*yC*_*ℓ*_*y*)⊆∩_*ℓ*=1_^*n*^(*zC*_*ℓ*_*z*) for all *y*∈∩_*ℓ*=1_^*n*^(*zC*_*ℓ*_*z*). Consequently, ∩_*ℓ*=1_^*n*^(*yC*_*ℓ*_*y*)⊆*M* for all *y*∈∩_*ℓ*=1_^*n*^(*zC*_*ℓ*_*z*) and so *y*∈_(*n*)_*E*_⊗_*L*(*M*) for all *y*∈∩_*ℓ*=1_^*n*^(*zC*_*ℓ*_*z*). Hence, ∩_*ℓ*=1_^*n*^(*zC*_*ℓ*_*z*)⊆ _(*n*)_*E*_⊗_*L*(*M*). So, *z*∈_(*n*)_*E*_⊗_*L*(_(*n*)_*E*_⊗_*L*(*M*)), which implies that _(*n*)_*E*_⊗_*L*(*M*)⊆_(*n*)_*E*_⊗_*L*(_(*n*)_*E*_⊗_*L*(*M*)) and so _(*n*)_*E*_⊗_*L*(*M*) = _(*n*)_*E*_⊗_*L*(_(*n*)_*E*_⊗_*L*(*M*)).

Mostly, the opposite of (4) of Proposition 8 cannot be valid, as we show in the next example.

**Example 3 .**

Consider ℛ_1_, ℛ_2_ are two binary relations on Λ = {*β*_1_, *β*_2_, *β*_3_, *β*_4_} such that

 
ℛ_1_ = {(*β*_1_, *β*_1_), (*β*_2_, *β*_2_), (*β*_2_, *β*_4_), (*β*_3_, *β*_1_), (*β*_4_, *β*_2_), (*β*_4_, *β*_4_)}
 
ℛ_2_ = {(*β*_1_, *β*_1_), (*β*_1_, *β*_2_), (*β*_2_, *β*_2_), (*β*_2_, *β*_3_), (*β*_2_, *β*_4_), (*β*_3_, *β*_3_), (*β*_4_, *β*_3_)}

If *M* = {*β*_2_}, $\overset{´}{M}$ = {*β*_4_}, then _(2)_*E*_⊗_*L*(*M*) = ∅, ${}_{\left(2\right)}E_{⊗}L\left(\overset{´}{M}\right)$ = {*β*_4_}, ${}_{\left(2\right)}E_{⊗}L\left(M\cup \overset{´}{M}\right)$ = {*β*_2_, *β*_4_}. So, ${}_{\left(2\right)}E_{⊗}L\left(M\cup \overset{´}{M}\right)\neq_{\left(2\right)}E_{⊗}L\left(M\right)\cup_{\left(2\right)}E_{⊗}L\left(\overset{´}{M}\right)$.

In view of Example 3, the converse of 5, 7 Proposition 3 does not hold. If *M*={*β*_2_}, $\overset{´}{M}=\left\{\beta_{4}\right\}$, then ${}_{\left(2\right)}E_{⊗}L\left(M\right)\subseteq_{\left(2\right)}E_{⊗}L\left(\overset{´}{M}\right)$, but $M⊈\overset{´}{M}$. Also, $M⊈{\overset{´}{M}}_{\left(n\right)}E_{⊗}L\left(M\right)$.

**Proposition 9 .**

If each ℛ_*ℓ*_, *ℓ* ∈ {1,2,…, *n*} is a dominance relation on Λ and *M*⊆Λ, then the coming statements hold:

1. *M*⊆_(*n*)_*E*_⊗_*L*(^(*n*)^*E*_⊗_*U*((*M*))
2. *M*⊇^(*n*)^*E*_⊗_*U*(_(*n*)_*E*_⊗_*L*(*M*))
3. ^(*n*)^*E*_⊗_*U*(*M*) = _(*n*)_*E*_⊗_*L*(^(*n*)^*E*_⊗_*U*(*M*))
4. _(*n*)_*E*_⊗_*L*(*M*) = ^(*n*)^*E*_⊗_*U*(_(*n*)_*E*_⊗_*L*(*M*))

**Proof**

1. Let *x* ∈ *M*. We shall prove that ∩_*ℓ*=1_^*n*^(*xC*_*ℓ*_*x*)⊆^(*n*)^*E*_⊗_*U*(*M*), i.e., ∩_*ℓ*=1_^*n*^(*yC*_*ℓ*_*y*)∩*M* ≠ ∅, ∀*y*∈∩_*ℓ*=1_^*n*^(*xC*_*ℓ*_*x*). In view of 7^•^ of Proposition 3, *x*∈^(*n*)^*E*_⊗_*U*(*M*). Let *y* ∈ ∩_*ℓ*=1_^*n*^(*xC*_*ℓ*_*x*). Since each ℛ_*ℓ*_, *ℓ* ∈ {1,2,…, *n*} is a dominance relation on Λ, then by Corollary 3, ∩_*ℓ*=1_^*n*^(*yC*_*ℓ*_*y*)∩*M*=∩_*ℓ*=1_^*n*^(*xC*_*ℓ*_*x*)∩*M* ≠ ∅. So the required has proven.
2. Similar to 1.
3. Obviously, from 7 of Proposition 8, _(*n*)_*E*_⊗_*L*(^(*n*)^*E*_⊗_*U*(*M*))⊆^(*n*)^*E*_⊗_*U*(*M*). On the other hand, in view of 8^•^ of Proposition 8 and 1 of this proposition, ^(*n*)^*E*_⊗_*U*(*M*)⊆_(*n*)_*E*_⊗_*L*(^(*n*)^*E*_⊗_*U*(^(*n*)^*E*_⊗_*U*(*M*))) = _(*n*)_*E*_⊗_*L*(^(*n*)^*E*_⊗_*U*(*M*)). So, ^(*n*)^*E*_⊗_*U*(*M*) = _(*n*)_*E*_⊗_*L*(^(*n*)^*E*_⊗_*U*(*M*)).
4. Similar to 3.

The next example shows that the statements 1 and 3 of Proposition 9 do not hold for any relation ℛ_*ℓ*_, *ℓ* ∈ {1,2,…, *n*} on Λ, except the dominance (resp. equivalence) relation.

**Example 4 .**

If Λ = {*β*_1_, *β*_2_, *β*_3_, *β*_4_} and △ is an identity relation on Λ, then we discuss the following cases:

1. Suppose ℛ_1_, ℛ_2_ are two binary relations on Λ defined as ℛ_1_ = {(*β*_1_, *β*_1_), (*β*_1_, *β*_2_), (*β*_1_, *β*_3_), (*β*_1_, *β*_4_), (*β*_2_, *β*_3_), (*β*_2_, *β*_4_), (*β*_3_, *β*_2_), (*β*_3_, *β*_4_), (*β*_4_, *β*_1_), (*β*_4_, *β*_2_), (*β*_4_, *β*_3_), (*β*_4_, *β*_4_)}ℛ_2_ = {(*β*_1_, *β*_1_), (*β*_1_, *β*_4_), (*β*_2_, *β*_2_), (*β*_3_, *β*_1_), (*β*_3_, *β*_2_), (*β*_3_, *β*_4_), (*β*_4_, *β*_1_), (*β*_4_, *β*_4_)}Then we get ∩_*ℓ*=1_^2^(*β*_1_*C*_*ℓ*_*β*_1_) = {*β*_1_}, ∩_*ℓ*=1_^2^(*β*_2_*C*_*ℓ*_*β*_2_) = {*β*_2_}, ∩_*ℓ*=1_^2^(*β*_3_*C*_*ℓ*_*β*_3_) = {*β*_3_}, ∩_*ℓ*=1_^2^(*β*_4_*C*_*ℓ*_*β*_4_) = {*β*_1_, *β*_4_}.
2. If *M* = {*β*_4_}, then ^(2)^*E*_⊗_*U*(*M*) = {*β*_4_}, _(2)_*E*_⊗_*L*(^(2)^*E*_⊗_*U*(*M*)) = ∅.
3. Suppose ℛ_1_, ℛ_2_ are two reflexive relations on Λ defined as ℛ_1_ = △∪{(*β*_1_, *β*_2_), (*β*_1_, *β*_4_), (*β*_3_, *β*_4_), (*β*_4_, *β*_3_)} and ℛ_2_ = △∪{(*β*_1_, *β*_3_), (*β*_1_, *β*_4_), (*β*_2_, *β*_3_), (*β*_2_, *β*_4_), (*β*_3_, *β*_4_), (*β*_4_, *β*_3_)}.
4. ∩_*ℓ*=1_^2^(*β*_1_*C*_*ℓ*_*β*_1_) = {*β*_1_}, ∩_*ℓ*=1_^2^(*β*_2_*C*_*ℓ*_*β*_2_) = {*β*_2_}, ∩_*ℓ*=1_^2^(*β*_3_*C*_*ℓ*_*β*_3_) = {*β*_3_}, ∩_*ℓ*=1_^2^(*β*_4_*C*_*ℓ*_*β*_4_) = {*β*_3_, *β*_4_}.
5. If *M* = {*β*_4_}, then ^(2)^*E*_⊗_*U*(*M*) = {*β*_4_}, _(2)_*E*_⊗_*L*(^(2)^*E*_⊗_*U*(*M*)) = ∅.
6. Suppose ℛ_1_, ℛ_2_ are two tolerance relations on Λ defined as ℛ_1_ = △∪{(*β*_1_, *β*_3_), (*β*_3_, *β*_1_), (*β*_3_, *β*_2_), (*β*_2_, *β*_3_), (*β*_4_, *β*_2_), (*β*_2_, *β*_4_), (*β*_4_, *β*_3_), (*β*_3_, *β*_4_)} and ℛ_2_ = △∪{(*β*_1_, *β*_2_), (*β*_2_, *β*_1_), (*β*_1_, *β*_3_), (*β*_3_, *β*_1_), (*β*_2_, *β*_3_), (*β*_3_, *β*_2_), (*β*_4_, *β*_2_), (*β*_2_, *β*_4_), (*β*_4_, *β*_3_), (*β*_3_, *β*_4_)}.
7. ∩_*ℓ*=1_^2^(*β*_1_*C*_*ℓ*_*β*_1_) = {*β*_1_}, ∩_*ℓ*=1_^2^(*β*_2_*C*_*ℓ*_*β*_2_) = {*β*_2_, *β*_4_}, ∩_*ℓ*=1_^2^(*β*_3_*C*_*ℓ*_*β*_3_) = Λ, ∩_*ℓ*=1_^2^(*β*_4_*C*_*ℓ*_*β*_4_) = {*β*_4_}.
8. If *M* = {*β*_1_, *β*_2_}, we find that ^(2)^*E*_⊗_*U*(*M*) = {*β*_1_, *β*_2_, *β*_3_}, _(2)_*E*_⊗_*L*(^(2)^*E*_⊗_*U*(*M*)) = {*β*_1_}.
9. Suppose ℛ_1_, ℛ_2_ are two symmetric and transitive relations on Λ defined as ℛ_1_ = {(*β*_2_, *β*_3_), (*β*_3_, *β*_2_), (*β*_2_, *β*_1_), (*β*_1_, *β*_2_), (*β*_1_, *β*_1_), (*β*_2_, *β*_2_), (*β*_3_, *β*_3_)}ℛ_2_ = {(*β*_1_, *β*_1_), (*β*_2_, *β*_2_), (*β*_3_, *β*_3_), (*β*_1_, *β*_3_), (*β*_1_, *β*_2_), (*β*_3_, *β*_1_), (*β*_2_, *β*_1_), (*β*_2_, *β*_3_), (*β*_3_, *β*_2_)}
10. ∩_*ℓ*=1_^2^(*β*_1_*C*_*ℓ*_*β*_1_) = {*β*_1_, *β*_4_}, ∩_*ℓ*=1_^2^(*β*_2_*C*_*ℓ*_*β*_2_) = Λ, ∩_*ℓ*=1_^2^(*β*_3_*C*_*ℓ*_*β*_3_) = {*β*_3_, *β*_4_}, ∩_*ℓ*=1_^2^(*β*_4_*C*_*ℓ*_*β*_4_) = {*β*_4_}
11. If *M* = {*β*_1_, *β*_3_}, we find that ^(2)^*E*_⊗_*U*(*M*) = {*β*_1_, *β*_2_, *β*_3_}, _(2)_*E*_⊗_*L*(^(2)^*E*_⊗_*U*(*M*)) = ∅.

**Remark 4 .**

According to the statements 2, 3, 7, and 8 of Proposition 8, the operator _(*n*)_*E*_⊗_*L*(.) satisfies Kuratowski's interior axioms and so one can deduce a topology ^(*n*)^⊤ on Λ that is given by ^(*n*)^⊤ = {*M*⊆Λ :  _(*n*)_*E*_⊗_*L*(*M*)=*M*}.

Next, we have the following remark using Corollary 5.

**Remark 5 .**

If each ℛ_*ℓ*_, *ℓ* ∈ {1,2,…, *n*} is a dominance relation on Λ, then ∩_*ℓ*=1_^*n*^*zC*_*ℓ*_*z* is ^(*n*)^⊤ open set for each *z* ∈ Λ.

**Theorem 1 .**

Let Λ be a universe and *z* ∈ Λ, then the coming statements hold:

1. If every ℛ_*ℓ*_, *ℓ* ∈ {1,2,…, *n*} is a reflexive relation on Λ, then the topology ^(*n*)^⊤_*C*_*ℓ*__ produced from *zC*_*ℓ*_*z* is finer than the topology ^(*n*)^⊤_ℛ_*ℓ*__ generated by *z*ℛ_*ℓ*_*z*
2. If every ℛ_*ℓ*_, *ℓ* ∈ {1,2,…, *n*} is a transitive relation on Λ, then the topology ^(*n*)^⊤_ℛ_*ℓ*__ produced from *z*ℛ_*ℓ*_*z* is finer than the topology ^(*n*)^⊤_*C*_*ℓ*__ generated by *zC*_*ℓ*_*z*
3. If every ℛ_*ℓ*_, *ℓ* ∈ {1,2,…, *n*} is a dominance relation on Λ, then the topology ^(*n*)^⊤_ℛ_*ℓ*__ produced from *z*ℛ_*ℓ*_*z* and the topology ^(*n*)^⊤_*C*_*ℓ*__ generated by *zC*_*ℓ*_*z* coincide

**Example 5 .**

If △ is an identity relation defined on Λ = {*β*_1_, *β*_2_, *β*_3_, *β*_4_}, then we discuss the following cases:

Suppose ℛ_1_, ℛ_2_ are two reflexive relations on Λ defined as Example 1. Then,

 
^(*n*)^⊤_ℛ_*ℓ*__ = {Λ, ∅, {*β*_1_}, {*β*_2_}, {*β*_1_, *β*_2_}, {*β*_3_, *β*_4_}, {*β*_1_, *β*_3_, *β*_4_}, {*β*_2_, *β*_3_, *β*_4_}}
 
^(*n*)^⊤_*C*_*ℓ*__ = {Λ, ∅, {*β*_1_}, {*β*_2_}, {*β*_4_}, {*β*_1_, *β*_2_}, {*β*_1_, *β*_4_}, {*β*_2_, *β*_4_}, {*β*_3_, *β*_4_}, {*β*_1_, *β*_2_, *β*_4_}, {*β*_1_, *β*_3_, *β*_4_}, {*β*_2_, *β*_3_, *β*_4_}}
 
^(*n*)^⊤_〈*C*_*ℓ*_〉_ = ^(*n*)^⊤_〈ℛ_*ℓ*_〉_ = {Λ, ∅, {*β*_1_}, {*β*_2_}, {*β*_3_}, {*β*_1_, *β*_2_}, {*β*_1_, *β*_3_}, {*β*_2_, *β*_3_}, {*β*_3_, *β*_4_}, {*β*_1_, *β*_2_, *β*_3_}, {*β*_1_, *β*_3_, *β*_4_}, {*β*_2_, *β*_3_, *β*_4_}}

Suppose ℛ_1_, ℛ_2_ are two transitive relations on Λ defined as

 
ℛ_1_ = {(*β*_1_, *β*_4_), (*β*_2_, *β*_1_), (*β*_2_, *β*_2_), (*β*_2_, *β*_3_), (*β*_2_, *β*_4_), (*β*_3_, *β*_1_), (*β*_3_, *β*_4_)}
 
ℛ_2_ = {(*β*_1_, *β*_1_), (*β*_1_, *β*_4_), (*β*_2_, *β*_1_), (*β*_2_, *β*_2_), (*β*_2_, *β*_3_), (*β*_2_, *β*_4_), (*β*_3_, *β*_1_), (*β*_3_, *β*_4_), (*β*_4_, *β*_4_)}
 
∩_*ℓ*=1_^2^(*β*_1_ℛ_*ℓ*_*β*_1_) = ∅, ∩_*ℓ*=1_^2^(*β*_2_ℛ_*ℓ*_*β*_2_) = {*β*_2_}, ∩_*ℓ*=1_^2^(*β*_3_ℛ_*ℓ*_*β*_3_) = ∅, ∩_*ℓ*=1_^2^(*β*_4_ℛ_*ℓ*_*β*_4_) = ∅
 
∩_*ℓ*=1_^2^(*β*_1_*C*_*ℓ*_*β*_1_) = {*β*_1_}, ∩_*ℓ*=1_^2^(*β*_2_*C*_*ℓ*_*β*_2_) = {*β*_2_, *β*_3_}, ∩_*ℓ*=1_^2^(*β*_3_*C*_*ℓ*_*β*_3_) = {*β*_3_}, ∩_*ℓ*=1_^2^(*β*_4_*C*_*ℓ*_*β*_4_) = {*β*_4_}
 
∩_*ℓ*=1_^2^(ℛ_*ℓ*_〈*β*_1_〉ℛ_*ℓ*_) = ∩_*ℓ*=1_^2^(*C*_*ℓ*_〈*β*_1_〉*C*_*ℓ*_) = {*β*_1_}, ∩_*ℓ*=1_^2^(ℛ_*ℓ*_〈*β*_2_〉ℛ_*ℓ*_) = ∩_*ℓ*=1_^2^(*C*_*ℓ*_〈*β*_2_〉*C*_*ℓ*_) = {*β*_2_}
 
∩_*ℓ*=1_^2^(ℛ_*ℓ*_〈*β*_3_〉ℛ_*ℓ*_) = ∩_*ℓ*=1_^2^(*C*_*ℓ*_〈*β*_3_〉*C*_*ℓ*_) = {*β*_2_, *β*_3_}, ∩_*ℓ*=1_^2^(ℛ_*ℓ*_〈*β*_4_〉ℛ_*ℓ*_) = ∩_*ℓ*=1_^2^(*C*_*ℓ*_〈*β*_4_〉*C*_*ℓ*_) = {*β*_4_}
 
^(*n*)^⊤_ℛ_*ℓ*__ = *P*(Λ)
 
^(*n*)^⊤_*C*_*ℓ*__ = {Λ, ∅, {*β*_1_}, {*β*_3_}, {*β*_4_}, {*β*_1_, *β*_3_}, {*β*_1_, *β*_4_}, {*β*_2_, *β*_3_}, {*β*_3_, *β*_4_}, {*β*_1_, *β*_2_, *β*_3_}, {*β*_1_, *β*_3_, *β*_4_}, {*β*_2_, *β*_3_, *β*_4_}}
 
^(*n*)^⊤_〈*C*_*ℓ*_〉_ = ^(*n*)^⊤_〈ℛ_*ℓ*_〉_ = {Λ, ∅, {*β*_1_}, {*β*_2_}, {*β*_4_}, {*β*_1_, *β*_2_}, {*β*_1_, *β*_4_}, {*β*_2_, *β*_3_}, {*β*_2_, *β*_4_}, {*β*_1_, *β*_2_, *β*_3_}, {*β*_1_, *β*_3_, *β*_4_}, {*β*_2_, *β*_3_, *β*_4_}}

**Remark 6 .**

According to Example 5,

(1) The converse of 1 and 2 of Theorem 1 may not be true in general, i.e.,
  - If each ℛ_*ℓ*_, *ℓ* ∈ {1,2,…, *n*} is reflexive, ^(*n*)^⊤_*C*_*ℓ*__⊈^(*n*)^⊤_ℛ_*ℓ*__
  - If each ℛ_*ℓ*_, *ℓ* ∈ {1,2,…, *n*} is transitive, ^(*n*)^⊤_ℛ_*ℓ*__⊈^(*n*)^⊤_*C*_*ℓ*__
(2) ^(*n*)^⊤_〈*C*_*ℓ*_〉_, ^(*n*)^⊤_*C*_*ℓ*__ are not comparable
(3) ^(*n*)^⊤_〈ℛ_*ℓ*_〉_, ^(*n*)^⊤_*C*_*ℓ*__ are not comparable
(4) If each ℛ_*ℓ*_, *ℓ* ∈ {1,2,…, *n*} is reflexive, then ^(*n*)^⊤_ℛ_*ℓ*__⊆^(*n*)^⊤_〈ℛ_*ℓ*_〉_
(5) If each ℛ_*ℓ*_, *ℓ* ∈ {1,2,…, *n*} is transitive, then ^(*n*)^⊤_〈ℛ_*ℓ*_〉_⊆^(*n*)^⊤_ℛ_*ℓ*__

## 4. Proposed Feature Selection Method

In this section, the proposed FS method is based on HBO and CRSA. However, the basic steps of the HBO are introduced in Section 4.1. Then, we discuss the developed method.

### 4.1. Honey Badger Optimization Algorithm

In this section, the mathematical notation of honey badger optimization algorithm is introduced. In general, HBO emulates the behaviour of honey badger to catch its prey. This process is performed through a set of stages named Digging and Honey. In the Digging stage, the prey is determined based on the smelling of honey badger, whereas in the Honey stage, the honey badger follows the honey bird for determining the beehive.

The steps of HBO begin by setting the initial of value of agents using the following:

(5) $$\begin{array}{c} Z_{i}=LB_{i}+r_{1}*\left(UB_{i}-LB_{i}\right),\;i=1,2,\ldots ,N, \end{array}$$

where **LB** is the lower boundary and **UB** is the upper boundary of the search space. *r*_1_ ∈ [0,1] refers to a random number. Followed by [57], the exploration (Digging) and exploitation (Honey) are balanced using the density factor (*α*) that is defined as

(6) $$\begin{array}{c} \alpha =C*\exp^{\left(-t/T\right)}, \end{array}$$

where *C* > 1 stands for a constant value, *T* represents the total number of iterations, and *t* indicates the current iteration.

The next step is to update the solutions using the operators of Digging stage. This is performed based on the cardioid movements formulated as

(7) $$\begin{array}{c} Z^{\text{new}}=Z_{b}+F*\beta *I*Z_{b}+F*\alpha *d^{i}*r_{3}*\left|\cos\left(2\pi r_{4}\right)*\left[1-\cos\left(2\pi r_{5}\right)\right]\right|. \end{array}$$

In equation (7), **Z**^new^ stands for the new value of **Z**_*i*_, **Z**_*b*_ represents the best solution found so far, and *r*_3_, *r*_4_, *r*_5_, and *r*_6_ are random numbers. (*B*) is a constant number. (*F*) is a parameter used to control the search direction and it is the value determined using the following equation:

(8) $$\begin{array}{c} F=\left\{\begin{array}{cc} 1, & \text{If }\left(r_{6}\leq 0.5\right),\\ -1, & \text{Else}. \end{array}\right. \end{array}$$

*I* stands for the smell intensity of the prey (*x*_*b*_) and it is used to represent the distance between the *x*_*b*_ and *x*_*i*_. It is formulated as

(9) $$\begin{array}{c} I_{i}=r_{2}*\frac{S}{4\pi d_{i}^{2}}, \end{array}$$

(10) $$\begin{array}{c} S={\left(Z^{i}-Z^{i+1}\right)}^{2},d_{i}=Z_{b}-Z_{i}. \end{array}$$

Meanwhile, the solutions can be updated using operators of Honey stage. This process is achieved using the following formula:

(11) $$\begin{array}{c} Z^{\text{new}}=Z_{b}+F*r_{7}*\alpha *d_{i}, \end{array}$$

where *r*_7_ is a random number. The steps of HBO are given in Algorithm 1.

### 4.2. Proposed HBOCRSA Framework

**Figure 1:**
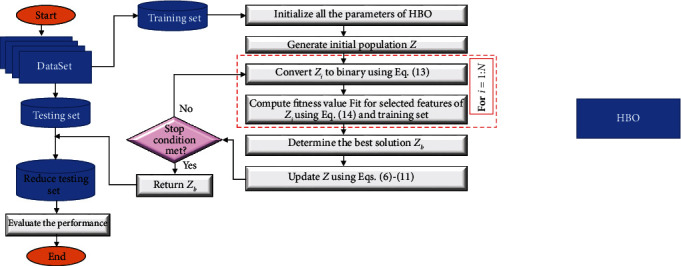
Steps of FS method based on the developed HBOCRSA technique.

We present the steps of the developed feature selection based on modified HBO and combined it with CRSA approximations in this section.  Figure 1 depicts the enhanced method structure. The main aim of the binary HBO (BHBO) algorithm based on CRSA (HBOCRSA) is identifying the subset of features that are more relevant regarding the dependencies degree among the features with the target features. As well as to deal with the discrete FS problem, binary HBO is applied.

The initialization of HBOCRSA is represented in identifying the features number (*f*_*k*_,  *k*=1,2,…, *d*) of the dataset corresponding to each solution dimension. Then, such dataset is split into training and testing sets. As well as, through the interval [0, 1], the generation of the initial value for *N* agents **Z** is performed. Furthermore, the computation of the value of the objective function of **Z**_*i*_,  *i*=1,2,…, *N* with determining the finest among them is performed. Through utilizing the HBO operators, modernize the agents. The modernization process is repeated till the stopping condition is met. Each phase is discussed in detail as follows.

#### 4.2.1. First Stage: Generating Solutions

The dataset is split into training and testing sets, by the percentages 80% and 20%, respectively, through the enhanced FS method. The *N* agents **Z** are initialized by the following equation:

(12) $$\begin{array}{c} Z_{i,j}=LB_{j}+r_{1}\times \left(UB_{j}-LB_{j}\right),\;i=1,2,\ldots ,N,\;j=1,2,\ldots ,D. \end{array}$$

In equation (12), **UB**_*j*_ and **LB**_*j*_ exemplify the dimension *j* upper and lower boundaries.

#### 4.2.2. Second Stage: Updating Solutions

The proposed technique is initialized through computing, for each agent **Z**_*i*_, the objective function value for two iterations.

The main goal of the first iteration is transferring **Z**_*i*_ into binary, as shown in

(13) $$\begin{array}{c} BZ_{ij}=\left\{\begin{array}{cc} 1, & \text{if }Z_{ij}>0.5,\\ 0, & \text{otherwise}. \end{array}\right.. \end{array}$$

Such process is used for transferring the real-valued agents to discrete ones which can deal with making them the feature selection problem.

The second step in the developed method contains choosing the features that are more relevant regarding ones in **BZ** and at the same time deleting the irrelevant features that correspond to the zeros. Furthermore, the quality first features are evaluated based on the function (Fit_*i*_) given as in equation (14). This is considered as the optimization problem which aims to minimize the error classification and minimize the number of features.

(14) $$\begin{array}{c} \text{Fit}\left(Z_{i}\right)=\eta \times \frac{\left|Z_{i}\right|}{d}+ℛ\times \left(1-\gamma_{C}\left(D\right)\right),ℛ+\eta =1, \end{array}$$

where |**Z**_*i*_| exemplifies the features number, as in equation (14), which is chosen by utilizing the **Z**_*i*_ current value. In addition, *d* exemplifies the number of features inside the dataset. ℛ and *η* are the coefficients that have the ability for balancing the number of selected features with the dependency degree *γ*_*C*_(*D*) given by

(15) $$\begin{array}{c} \gamma_{C}\left(D\right)=\frac{\left|POS_{C}\left(D\right)\right|}{\left|U\right|}. \end{array}$$

In equation (15), *POS*_*C*_(*D*) represents the positive region defined as

(16) $$\begin{array}{c} POS_{C}\left(D\right)=\cup \underline{B}\left(Z\right),Z\in \frac{U}{D}, \end{array}$$

where $\underline{B}\left(Z\right)$ exemplifies the lower approximation given in equation (1). In addition, *γ*_*C*_(*D*) aims for computing the features approximating power.

Finally, the third step aims for identifying the finest agent *Z*_*b*_, after that the agents are modernized by utilizing the HBO operators given in Section 4.1.

#### 4.2.3. Third Stage: Stop Conditions

Meeting the stopping condition for the proposed technique can be tested and returning the finest solution as the solutions accepted with repeating the strides existed in the second stage. Moreover, the testing set is used to assess the relevant features existed in the finest solution *Z*_*b*_ and perform classification for the reduced set by utilizing the KNN classifier for the features quality assessing.

## 5. Experimental Results and Discussion Using Real-World Datasets

The performance of the developed method can be justified through the modified CRSA which is considered as RS extension with concluding some of the experiments. Through our work and experiments, we depended on using ten datasets, in addition to comparing the results with other FS approaches. In this experiment, the proposed BHBA is used to determine the relevant feature from the given data. To conduct this, the CRSA is used as a part of the fitness function that is defined in equation (14).

### 5.1. Datasets Description

The quality of HBOCRSA is validated by utilizing ten datasets with varying dimensionality. The datasets, gathered from various fields, are taken online from UCI [58], and those ones that we depended in our work are shown in Table 1 where they have various instance numbers, feature numbers, and classes.

The efficiency of HBOCRSA can be validated and justified, where the dataset is split into 80% and 20% training and testing sets, respectively, where these percentages form the whole number. Through 30 independent times, each algorithm has been conducted for guaranteeing the comparison quality. The comparisons are performed through using the algorithms of the salp swarm algorithm (SSA), self-adaptive differential evolution (SaDE), grey-wolf optimization (GWO), genetic algorithm (GA), and teaching-learning-based optimization (TLBO) as competing ones for feature selection. With taking the note that each algorithm parameter is set regarding its implementation, the iteration number and the population size are set to 15 and 20 which are considered the common parameters. Thereafter, each solution in the population has dimension which equals to the features number for each dataset.

### 5.2. Performance Measures

More metrics for the proposed technique, HBOCRSA, assessing can be utilized. Some of such measures or metrics are accuracy and the selected features number; such measures can be defined as

(i) Average accuracy (AVG_Acc_) exemplifies the accuracies average; overall, the runs number (*N*_*r*_=30) is given as
  (17) $$\begin{array}{c} {\text{AVG}}_{\text{Acc}}=\frac{1}{N_{r}}\sum_{k=1}^{N_{r}}{\text{Acc}}_{\text{Best}}^{k}, \end{array}$$
  (18) $$\begin{array}{c} {\text{Acc}}_{\text{Best}}^{k}=\frac{\text{TP}+\text{TN}}{\text{TP}+\text{FN}+\text{FP}+\text{TN}}. \end{array}$$
(ii) Average number of the selected features (AVG_*|BZ*_Best_*|*_) calculates the features average chosen by each algorithm through all runs and it is given as
  (19) $$\begin{array}{c} {\text{AVG}}_{\left|{\text{BZ}}_{\text{Best}}\right|}=\frac{1}{N_{r}}\sum_{k=1}^{N_{r}}\left|{\text{BZ}}_{\text{Best}}^{k}\right|. \end{array}$$

In equation (15), |BZ_Best_^*k*^| exemplifies the finest solution cardinality at *k*^*th*^ run.

### 5.3. Experimental Series 1: Results and Discussion Using UCI Datasets

**Figure 2:**
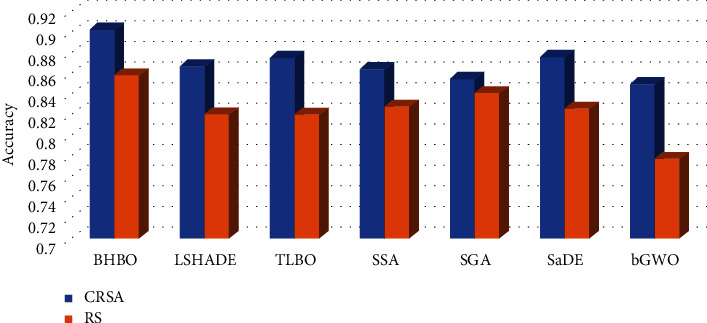
Average of accuracy of each algorithm among the tested datasets.

**Figure 3:**
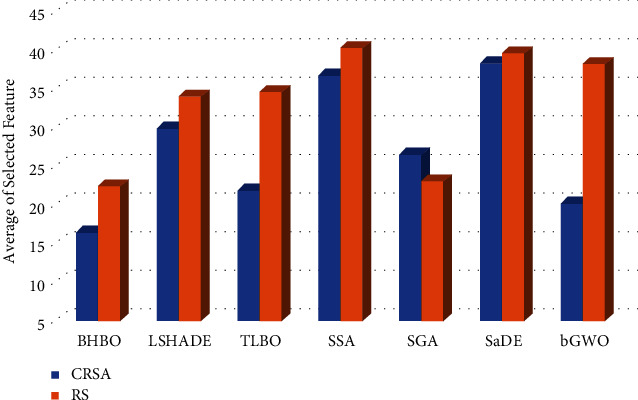
Average of selected features of each algorithm among the tested datasets.

The comparison results between the developed method and other competitive methods are given in Tables 2–5 and Figures 2 and 3. From these results, it can be noticed that the HBO using CRSA (i.e., HBOCRSA) has high accuracy at four datasets (i.e., D2-D4 and D8). However, for the other datasets, its results are competitive with other methods, for example, at D1, D7, D9, HBOCRSA allocates the second rank. Moreover, the average of each method among the tested datasets is given in Figure 2 and it can be noticed the difference between HBOCRSA and LSHADE, TLBO, SSA, SGA, SaDE, and bGWO is 3.527%, 2.711%, 3.799%, 4.754%, 2.638%, 5.258%, respectively. In case the traditional RS is used as part of fitness value (as in Table 3), HBORS provides the better accuracy at D2, D4, D7, and D9. In addition, its average overall, the tested ten datasets are better than other methods with difference 3.780%, 3.7918%, 3.009%, 1.6491%, 3.218%, and 8.0456% when compared with LSHADE, TLBO, SSA, SGA, SaDE, and bGWO, respectively (see Figure 2).

Besides the accuracy obtained by each FS approach using either RS or CRSA, it can be noticed that the accuracy of each method is increased using CRSA. This indicates that CRSA has ability to determine the relevant features better than traditional RS.

To further analysis the behaviour of HBO using RS and CRSA, its ability to reduce the number of features is computed, as given in Table 4. From these results, it can be seen that HBO based on traditional RS provides smallest number of features at 70% from the tested datasets (i.e., all datasets, except D2, D3, and D8), followed by GA which has the smallest number at three datasets. In terms of the average of selected features overall the tested sets, it can be noticed that the HBO and GA allocate the first and second ranks, respectively; however, the difference between them is not significant. Followed by LSHADE and TLBO, whereas, the worst algorithm is SSA which has the largest number of features.

In case of using CRSA, the number of selected features using HBO is still the smallest number nearly 16 features. This is followed by bGWO and LSHADE that allocate the second and third ranks, respectively. By comparing the performance of each algorithm in terms of number of selected features using traditional RS and CRSA, it can be observed that RS provides number of features larger than those CRSA. For example, HBO, LSHADE, TLBO SSA, SGA, SaDE, and bGWO based on RS provide nearly 6.07, 4.18, 12.86, 3.71, −3.344, 1.22, and 18.14 features, respectively, greater than using CRSA.

**Figure 4:**
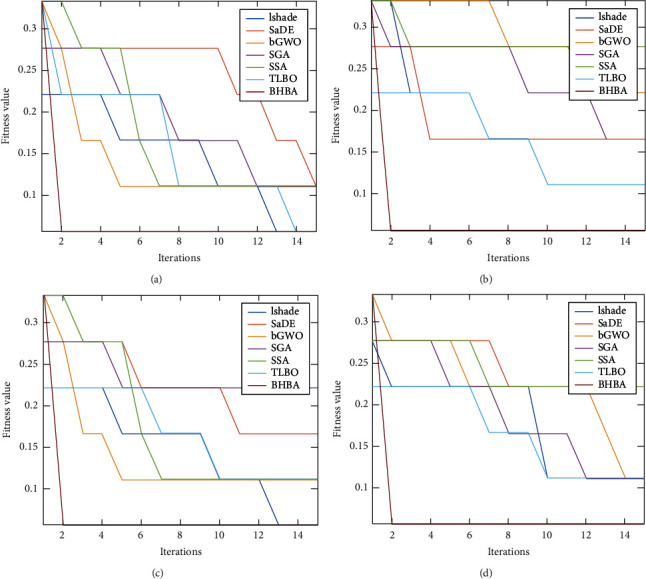
Convergence curve of the algorithms using RS as fitness value.

**Figure 5:**
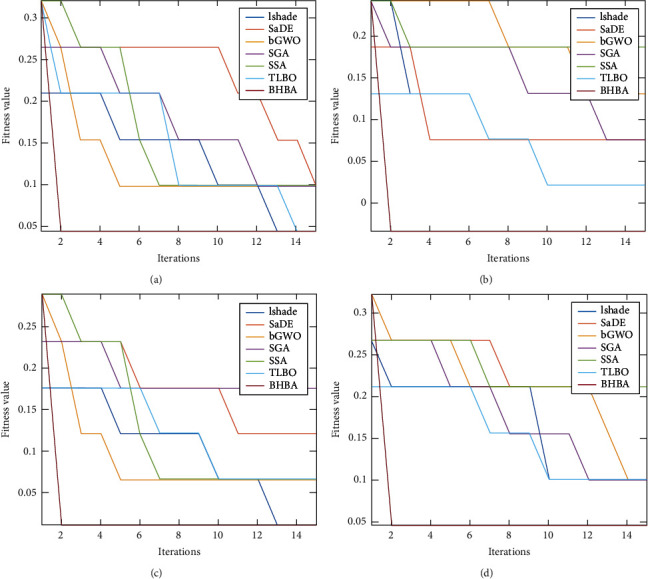
Convergence curve of the algorithms using CRSA as fitness value.

Figures 4 and 5 show an example of the average of convergence curve the first four dataset for FS algorithms using RS and CRSA, respectively, as fitness function. From these figures, it can be concluded that BHBA has high ability to converge faster than other methods in both cases RS and CRSA. In addition, by comparing the behaviour of the algorithm(s) using the CRSA, it can be noticed that they have ability better than using RS to minimize the fitness value.

To make a decision if the difference between the developed HBO using CRSA and other methods is significant or not, a nonparametric Friedman test is applied. The statistical values obtained using FR test are given in Table 5. From these values, it can be observed that the developed BHBO has the largest mean rank in terms of accuracy using both the traditional RS and new CRSA. In addition, the smallest mean rank in terms of number of selected features is achieved using BHBO. This indicates the combination between the BHBO and CRSA has been led to increase in the classification and decrease in the number of features.

It can be seen that the new HBOCRSA approach is more applicable and efficient than competing algorithms based on earlier evaluations utilizing UCI datasets. However, HBOCRSA still has significant drawbacks, such as a high computational complexity, especially when used to handle high-dimensional datasets. Using parallel processing and GPU hardware, this can be remedied. Furthermore, HBOCRSA's behaviour in balancing exploration and exploitation to find the viable region has the greatest impact on its performance. Combining it with additional operators can improve this.

## 6. Conclusion and Future Works

In this article, we focused on creating a novel model of rough set approximations (RSA), namely, the rough set approximation models depending on containment neighborhoods (CRSA), that generalize the classical notions of (RSA) and derive a number of distinguished results. To evaluate the appropriateness of this model, it is applied to enhance the classification of contrasting dataset by utilizing it an objective function for distinct feature selection approach. This has been carried out by applying the binary version of honey badger optimization algorithm (BHBO) as feature selection method. The effects of HBOCRSA are compared with different MH techniques, which involve GWO, LSHADE, SSA, GA, TLBO, and SaDE. A class of ten datasets is employed to assess the performance of the developed method. The experiential results clarified the high performance of the developed method as FS approach which owns accuracy better than other methods. Furthermore, the number of the selected features acquired utilizing the HBOCRSA is smaller than the other methods. Additionally, the accomplishment of the model of (CRSA) is better than classical (RS) approach that is based on the factors of performance measures. Based on the favourable consequences gained from the developed method, it can be applied in diverse scopes, for instance, cloud computing, image processing, and IoT applications. In addition, it can be reconstructed as multiobjective technique and used to several sets of reality multiobjective problems involving feature, selection, and engineering issues and others.

## Figures and Tables

**Algorithm 1:**
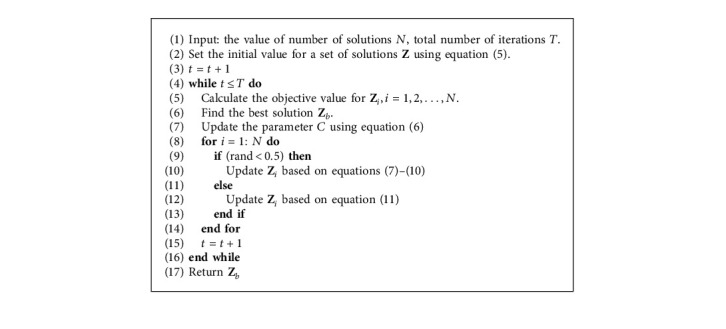
Steps of HBO.

**Table 1:** Description of datasets.

Datasets	No. of instances	No. of features	No. of classes	Data category
Breastcancer (D1)	699	9	2	Biology
BreastEW (D2)	569	30	2	Biology
CongressEW (D3)	435	16	2	Politics
Exactly2 (D4)	1000	13	2	Biology
HeartEW (D5)	270	13	2	Biology
IonosphereEW (D6)	351	34	2	Electromagnetic
Lymphography (D7)	148	18	2	Biology
PenglungEW (D8)	73	325	2	Biology
SonarEW (D9)	208	60	2	Biology
SpectEW (D10)	267	22	2	Biology

Bold values represent the best value.

**Table 2:** Accuracy of each algorithm using CRSA.

	BHBO	LSHADE	TLBO	SSA	SGA	SaDE	bGWO
D1	0.9705	0.9429	0.9571	0.9643	**0.9714**	0.9571	0.8929
D2	**0.9802**	0.9298	0.9561	0.9474	0.9386	0.9649	0.9211
D3	**0.9942**	0.9655	0.9333	0.9195	0.9425	0.9195	0.9655
D4	**0.9041**	0.6750	0.7500	0.6400	0.6850	0.6250	0.7500
D5	0.7465	0.7593	0.7778	0.7963	0.7963	**0.8333**	0.7778
D6	0.9294	0.8592	0.8592	0.8592	0.8451	0.8592	**0.9296**
D7	0.7803	0.7333	**0.8333**	0.7667	0.7333	0.8000	0.6667
D8	**1.0000**	0.9333	0.8000	0.9333	0.8667	0.9467	0.9333
D9	0.8786	**0.8810**	0.8571	**0.8810**	0.8333	**0.8810**	0.7619
D10	0.8376	0.8889	**0.9259**	0.8333	0.8333	0.8704	0.7963

**Table 3:** Accuracy of each algorithm using rough set.

	BHBO	LSHADE	TLBO	SSA	SGA	SaDE	bGWO
D1	0.9558	0.9386	0.9214	**0.9600**	0.9429	0.9514	0.9414
D2	**0.9420**	0.9211	0.9158	0.9158	0.9211	0.9263	0.9298
D3	0.9538	0.9494	0.8966	**0.9586**	0.9264	0.8920	0.9471
D4	**0.7897**	0.7180	0.7790	0.7040	0.7570	0.7270	0.7010
D5	0.7198	0.6889	**0.7741**	0.6889	0.7593	0.7111	0.6741
D6	0.8474	0.8563	0.8648	0.8873	**0.9211**	0.8986	0.8761
D7	**0.8364**	0.7933	0.7791	0.7163	0.7133	0.7667	0.6554
D8	0.8702	0.8200	0.7067	0.8667	0.8000	0.8667	0.5733
D9	**0.9060**	0.8190	0.8476	0.8619	0.8952	0.7952	0.7429
D10	0.7579	0.6963	0.7148	0.7185	**0.7778**	0.7222	0.7333

Bold values represent the best value.

**Table 4:** Number of selected features using RS and CRSA.

	BHBO		LSHADE		TLBO		SSA		GA		SaDE		bGWO	
	CRSA	RS	CRSA	RS	CRSA	RS	CRSA	RS	CRSA	RS	CRSA	RS	CRSA	RS
D1	1.8	**2.4**	**1.6**	2.8	2.1	3	3.5	3.8	**2.1**	3	2.8	3.6	3.4	2.8
D2	**6.5**	15.2	11.8	15.2	14.2	16	18.2	20.2	16.3	**8.4**	17.9	19.2	11.5	13
D3	**2.3**	7.3	6.2	7.2	5.9	8.2	8.2	10	7	**5.2**	6.9	8.8	3.9	6.4
D4	**2.4**	**8.2**	3.8	10.4	4.1	10.2	6.3	10.8	5.6	10	7	10.6	3.8	10.8
D5	**2.4**	**5.5**	3.4	6.4	3.8	7	6.2	7.8	5	6	7.1	7	4.4	6.6
D6	**7**	**14**	15.6	16.8	17.4	18.4	20.2	23	19.3	16.8	20.9	21	10.5	17.6
D7	**3.5**	**5.7**	6.6	8	6.9	9.6	8.1	11	7.9	9.8	8.9	10.4	4.8	6.2
D8	**112.4**	121.2	209	226	120	225.6	241	258.4	150.4	**117.6**	255.7	259	130.5	267
D9	**20.4**	**34.2**	32.2	36.4	34.2	36.6	41.5	44.4	40.8	42	42.4	42.4	21.4	41.2
D10	**4.7**	**10.4**	8.4	11.2	9.4	12	12.9	13.8	10.24	12.4	13.8	13.6	6.8	10.8

Bold values represent the best value.

**Table 5:** Mean rank for each algorithm using UCI datasets.

	BHBO	LSHADE	TLBO	SSA	SGA	SaDE	bGWO	*P* value
Accuracy using CRSA	5.10	3.65	4.20	4	3.45	4.55	3.05	0.3725
Accuracy using RS	5.90	3.20	3.35	4.15	4.65	4.05	2.70	0.0227
No. of features using CRSA	1.10	2.95	3.55	6.30	4.85	6.50	2.75	3.52e-09
No. of features using RS	1.65	3.05	4.20	6.75	2.90	5.85	3.60	3.22e-07

## Data Availability

The data used to support the findings of this study are available from the authors upon request.
